# Systemic Inflammation and Adverse Heart Failure Outcomes: Insights From the Veterans Affairs Health Care System

**DOI:** 10.1016/j.jacadv.2026.103346

**Published:** 2026-10-23

**Authors:** Alexander T. Sandhu, Adam Furst, Anubodh S. Varshney, Karim Sallam, Stephanie Hsiao, Anthony Gromovsky, Kat Wolf Khachatourian, Natasha Din, Jeffrey R. Skaar, Ivy Tonnu-Mihara

**Affiliations:** a Division of Cardiology, Department of Medicine, University of California Los Angeles, Los Angeles, California, USA; b Greater Los Angeles Veterans Affairs Healthcare System, Los Angeles, California, USA; c Palo Alto Veterans Affairs Healthcare System, Palo Alto, California, USA; d Division of Cardiology, Stanford University School of Medicine, Stanford, California, USA; e Novo Nordisk Inc., Plainsboro, New Jersey, USA

**Keywords:** heart failure with preserved ejection fraction, high-sensitivity C-reactive protein, inflammation, major adverse cardiovascular event

## Abstract

**Background:**

The association between systemic inflammation (SI) and outcomes among patients with heart failure with preserved ejection fraction (HFpEF) or mildly reduced ejection fraction (HFmrEF) is uncertain.

**Objectives:**

This study aimed to evaluate the association between SI and clinical and economic outcomes among individuals with HFpEF/HFmrEF.

**Methods:**

We identified Veterans with HFpEF/HFmrEF with outpatient high-sensitivity C-reactive protein (hsCRP) testing from 2008 to 2022. We classified Veterans as with SI (hsCRP 2-10 mg/L) and without SI (hsCRP <2 mg/L). The primary outcome was the 3-component major adverse cardiovascular event (MACE) outcome of death, HF hospitalization, and HF-related emergency department encounters. Additional outcomes included acute care utilization and costs. We evaluated the adjusted associations between SI and clinical outcomes, acute care utilization, and costs via Cox regression, negative binomial regression, and gamma regression, respectively.

**Results:**

We identified 49,986 Veterans with HFpEF/HFmrEF with outpatient hsCRP levels. We compared 8,696 (17.4%) Veterans without SI (hsCRP <2 mg/L) and 25,352 (50.7%) Veterans with SI (hsCRP ≥2 mg/L and ≤10 mg/L Over a median follow-up of 3.4 years (IQR: 1.8-5.8), MACE incidence was 15.1/100 patient-years (95% CI: 14.9-15.4) among those with SI vs 11.8/100 patient-years (95% CI: 11.4-12.1) among those without SI (*P* < 0.001). After adjustment, SI was associated with increased MACE (HR: 1.28; 95% CI: 1.24-1.33). SI was also associated with increased all-cause hospitalizations (incidence rate ratio: 1.19; 95% CI: 1.137-1.246) and $9,112 (95% CI: $6,135-$12,089) in additional per-patient costs over 1 year.

**Conclusions:**

Among Veterans with HFmrEF/HFpEF, SI is associated with increased adverse clinical outcomes and higher costs.

The prevalence of heart failure (HF) with preserved ejection fraction (HFpEF) or mildly reduced ejection fraction (HFmrEF) in the United States exceeds 3 million and continues to increase.^1^^,^^2^ HF is associated with substantial morbidity and mortality, and people with HFpEF/HFmrEF experience a risk of death comparable to those with HF with reduced ejection fraction (HFrEF).^3^, ^4^, ^5^

Systemic inflammation (SI) may be critical in the pathogenesis of HFpEF/HFmrEF.^6^^,^^7^ Each of the primary risk factors for HFpEF—chronic kidney disease, diabetes mellitus, hypertension, and obesity—increase SI.^8^ According to the emerging inflammatory hypothesis, ongoing SI promotes pathological remodeling in the heart with progressive hypertrophy and fibrosis.^6^^,^^7^^,^^9^^,^^10^ Prospective studies and clinical-trial substudies have shown that SI, reflected by elevated high-sensitivity C-reactive protein (hsCRP), is associated with worse outcomes in HFpEF/HFmrEF.^11^^,^^12^ However, these analyses have generally been limited to several 100 selected patients. Whether hsCRP measured during routine clinical practice predicts clinical and economic outcomes in a large, unselected HFpEF/HFmrEF population remains unknown.

The present analysis evaluated the prognostic significance of SI in the United States Veterans Health Administration (VHA)—the largest integrated health system in the nation. We compared clinical and economic outcomes among Veterans with HFpEF/HFmrEF who had elevated hsCRP levels vs those without elevated hsCRP between 2008 and 2022.

## Methods

### Data

We used electronic health record and administrative data from the VHA Corporate Data Warehouse, in addition to claims for Veterans’ purchased care and Medicare fee-for-service from 2005 through 2023. The VHA Corporate Data Warehouse includes demographics, outpatient and inpatient encounters, diagnoses, procedures, laboratory results, vital signs, and pharmacy dispensing records. Purchased care and Medicare claims data were used to identify clinical care and medication prescriptions provided to Veterans outside of the VHA health care system. Mortality data were based on the VHA Mortality Data Repository and the National Death Index. The underlying cause of death was available via National Death Index data through 2022.

### Study population and high-sensitivity C-reactive protein

We identified Veterans with HFpEF/HFmrEF based on left ventricular ejection fraction with an eligible outpatient hsCRP measurement between 2008 and 2022. We first identified hsCRP testing using Logical Observation Identifiers Names and Codes codes (Supplemental Table 1). We restricted the analysis to the first eligible hsCRP test performed in the outpatient setting in patients between 18 and 89 years of age with established HFpEF/HFmrEF at the time of testing. Because testing and results were analyzed retrospectively, we were unable to determine the specific clinical indication for each hsCRP order. HF was identified based on a previously validated approach with the presence of one prior principal inpatient HF diagnosis or at least 2 total HF diagnoses.^13^^,^^14^ Patients were classified by their most recent ejection fraction >40% within 3 years before the index hsCRP test and therefore could include patients with HF with improved ejection fraction (a prior left ventricular ejection fraction <40% with subsequent recovery to >40%). We included individuals with HF with improved ejection fraction given the persistent elevated risk of HF events in this cohort, and the evidence demonstrating benefit with therapies also shown to benefit patients with HFpEF.^15^, ^16^, ^17^, ^18^ In a sensitivity analysis, we repeated the primary analysis after excluding patients with left ventricular ejection fraction <40% during the lookback period. We identified ejection fraction based on a validated natural language processing algorithm that incorporates echocardiography reports and clinical notes.^19^ When ejection fraction data were unavailable from the natural language processing algorithm, we used previously validated International Classification of Diseases (ICD) codes (codes for diastolic HF) to identify HFmrEF/HFpEF.^20^

To identify a cohort with active VHA care, we excluded hsCRP tests without at least 1 diagnosis and clinical encounter in the preceding year. We also excluded tests in which the Veteran had active comorbidities in the preceding year that cause severe SI and increase the risk of adverse clinical outcome and health care costs: cancer receiving chemotherapy, metastatic cancer, HIV, or chronic osteomyelitis (Supplemental Table 1). These conditions were excluded to provide conservative estimates of the association between hsCRP and cardiovascular disease-associated clinical and economic outcomes, given these conditions mostly cause noncardiovascular morbidity and cost. Finally, we excluded tests within 60 days of myocardial infarction or stroke hospitalization given the transient rise in hsCRP levels after these events. In a sensitivity analysis, we also excluded tests among Veterans with established systemic autoimmune disease (Supplemental Table 1 for diagnosis codes). In a sensitivity analysis, we also excluded patients with a prescription for an antibiotic, antifungal, or antiviral within 30 days before or 14 days after the index hsCRP measurement.

For the primary analysis, we defined SI as hsCRP levels >2 mg/L and <10 mg/L. Prior analyses have used levels >2 mg/L as a benchmark for inflammatory risk.^21^^,^^22^ As has been done previously, we set an upper threshold of hsCRP <10 mg/L because marked elevation of hsCRP may be related to acute infections or other major systemic disorders with increased associated clinical morbidity and cost.^23^, ^24^, ^25^ As a sensitivity analysis, we repeated the analysis while including those with hsCRP >10 mg/L in the SI cohort.

Among Veterans with multiple eligible hsCRP tests, we applied a hierarchical selection rule to prioritize identification of Veterans with SI. We first identified hsCRP levels >2 mg/L and <10 mg/L at any point during the study period. For the remaining cohort, we selected the first eligible hsCRP measurement. This approach was used to expand the cohort with SI, although it modifies the estimate of individuals with SI at any time point. In a sensitivity analysis, we repeated the primary analysis restricted to Veterans with first hsCRP measurement. The date of the hsCRP test was set as the index date for evaluating outcomes.

### Study variables

We collected data on patients’ sociodemographic characteristics, vital signs, comorbidities, laboratory values, and medication use. Race and ethnicity were determined based on patient self-report. Vital signs were extracted from the most recent measurements available within the 3 years preceding the index date. Using each patient’s residential address, we assigned the corresponding Centers for Disease Control and Prevention Social Vulnerability Index, which quantifies community-level risk at the census tract level.^26^

We identified baseline clinical characteristics using a 3-year lookback period prior to hsCRP measurement. Medical comorbidities were identified using ICD-9 and ICD-10 diagnostic codes (see Supplemental Table 1). To identify frailty, we used a surrogate based on the presence of at least 2 diagnoses previously used as indicators of frailty in claims-based data.^27^ Cardiovascular procedures were captured using Current Procedural Terminology and ICD procedure codes (Supplemental Table 1). Laboratory values were extracted using Logical Observation Identifiers Names and Codes. Natriuretic peptide values are reported descriptively and separately as B-type natriuretic peptide and N-terminal pro-B-type natriuretic peptide levels among patients with each respective measurement; no conversion between the 2 assays was performed, and natriuretic peptides were not used in the adjusted models. We identified baseline medications based on a 180-day lookback period prior to hsCRP measurement. Medications commonly used for HFpEF/HFmrEF—glucagon-like peptide-1 receptor agonists, angiotensin-converting enzyme inhibitors/angiotensin receptor blockers/angiotensin receptor neprilysin inhibitors, anticoagulants, antihyperlipidemics, beta-blockers, diuretics, mineralocorticoid receptor antagonists, and sodium-glucose transport protein 2 inhibitors—were identified through prescription fill records from the VHA, Medicare Part D, and non-VHA medication entries documented in the electronic health record.

### Clinical outcomes

Our primary clinical outcome was a 3-component major adverse cardiovascular event (MACE) composite of all-cause death, HF hospitalization, and emergency department (ED) visits for HF. HF hospitalizations were acute care hospitalizations with a principal discharge diagnosis of HF. ED visits for HF were identified based on a primary diagnosis of HF. Secondary outcomes included the individual components of the primary outcome and cardiovascular death. Cardiovascular death was defined based on an underlying cause of death diagnosis code for cardiovascular disease (I.00-I.99) on death certificates (Supplemental Table 1).^28^^,^^29^ We selected an HF-specific composite outcome because the morbidity and mortality of HFpEF/HFmrEF are driven predominantly by HF events; as an exploratory secondary outcome, we also evaluated the composite outcome including myocardial infarction and stroke, which were identified based on hospitalizations with principal diagnoses of myocardial infarction and stroke, respectively. Follow-up began on the date of the index outpatient hsCRP measurement (index date) and continued until the earliest of the outcome of interest, the date of the last known clinical encounter within the VHA, Medicare, or purchased care (censoring for loss to follow-up), or the end of the study period on December 31, 2023. For the cardiovascular death outcome, follow-up was censored on December 31, 2022, given the availability of underlying cause of death data through 2022.

### Acute care utilization and health care costs

We evaluated health care utilization and costs to quantify whether SI is associated with increased economic burden in HFpEF/HFmrEF, providing insight into the broader health care impact beyond clinical outcomes alone. To assess acute care utilization, we quantified the frequency of all-cause hospitalizations, cardiovascular hospitalizations, HF hospitalizations, ED visits for HF, and ED visits for any reason. Cardiovascular hospitalizations were identified based on the principal discharge diagnosis, using ICD-9/ICD-10 codes for cardiovascular disease (ICD-10: I.00-I.99 and ICD-9: 390-459). We identified VHA ED visits based on the stop codes (a coding system used by the VHA that identifies the location of services) and non-VHA ED visits based on place of service codes. ED visits were restricted to those not resulting in admission.

We analyzed health care costs across several categories: total, inpatient, outpatient, pharmacy, and cardiovascular-related expenses. Cardiovascular-related costs were identified based on the presence of associated ICD codes indicative of cardiovascular disease (Supplemental Table 1). VHA costs were based on average cost methods in which services are weighted by Medicare relative value weights and adjusted to the total cost of care within the VHA.^30^ For Medicare and community care cost data, we used the total amount paid, including patient out-of-pocket costs. All costs were inflated to 2023 U.S. dollars via the Personal Health Care Index.^31^ Acute care utilization and cost outcomes were evaluated over fixed windows of 6 months, 1 year, 3 years, and 5 years following the index date, and each analysis was restricted to individuals with complete follow-up through the end of the respective window.

### Statistical analysis

We first described patient characteristics via means and SDs for continuous variables and counts and percentages for categorical variables, comparing characteristics between those with and without SI with a 2-sample *t*-test for normally distributed variables, Wilcoxon rank-sum for non-normally distributed values, and chi-squared for categorical variables.

For clinical outcomes, we compared incidence rates of each clinical outcome between those with and without SI. We evaluated the adjusted associations between SI and clinical outcomes using Cox regression models. We used cause-specific hazards, and we censored nonfatal events at the date of death. This approach addresses the etiologic association between SI and the rate of events among those at risk.^32^^,^^33^ For the cardiovascular death outcome, we also censored outcomes on December 31, 2022, given the lack of cause of death data for 2023. We repeated these analyses with alternate modeling of hsCRP. First, we modeled hsCRP via quartiles, with the lowest quartile as the reference category. Second, we modeled hsCRP as a continuous variable. Third, we modeled hsCRP as a restricted cubic spline with 4 knots placed at the 5th, 35th, 65th, and 95th percentiles of the observed distribution, as recommended by Harrell, and graphically displayed the association between hsCRP and the outcome. We assessed the proportional hazards assumption using Schoenfeld residuals. Given the global test demonstrated nonproportional hazards, the HRs are interpreted as time-averaged estimates, as has been suggested.

We modeled the association between SI and health care resource utilization using negative binomial regression models and displayed the results via incidence rate ratios (IRRs). Negative binomial models included an offset for the log of follow-up time to account for varying exposure. We modeled the association between SI and health care costs via gamma regression models, with the results displayed as the average marginal cost associated with SI. Given inpatient and cardiovascular costs are often zero, we modeled these cost categories via 2-stage models with a logit model for predicting any health care costs and the gamma model for predictions conditional on cost being greater than zero. In a sensitivity analysis, we repeated the cost analyses using Tobit regression models. We excluded individuals that were censored due to death or loss to follow-up prior to the end of the follow-up periods.

We used 2 nested adjusted models for all outcomes: (Model A) adjusted for age, sex, and year of hsCRP measurement and (Model B), which additionally adjusted for the ejection fraction category (EF ≥50% [HFpEF] vs EF >40% and EF<50% [HFmrEF]), Charlson Comorbidity Index, atherosclerotic cardiovascular disease, chronic kidney disease, smoking status, body mass index (BMI), estimated glomerular filtration rate, and medications with established benefit for HFpEF/HFmrEF (angiotensin-converting enzyme inhibitors/angiotensin receptor blockers/angiotensin receptor neprilysin inhibitors, glucagon-like peptide-1 receptor agonists, and sodium-glucose transport protein 2 inhibitors). We adjusted for these variables given the likelihood of association with clinical and economic outcomes and the exposure of interest, elevated hsCRP. Given the analysis focused on identifying the association between hsCRP and clinical and economic outcomes, we were not concerned about multicollinearity between other covariates. All continuous variables were modeled as restricted cubic splines with 4 knots placed at the 5th, 35th, 65th, and 95th percentiles of the observed distribution to account for nonlinearity. For patients with missing EF values, we imputed preserved ejection fraction, which is higher prevalence than mildly reduced ejection fraction. As detailed in the Supplemental Methods, missingness for BMI and estimated glomerular filtration rate were each below 6%; there were no other missing variables. Given the low rate of missingness, we used mean imputation and included missing indicators to account for potential nonrandom missingness.^34^ We additionally performed a complete case analysis of the primary clinical outcome as a sensitivity analysis.

We evaluated the association between SI and outcomes while adjusting for age (in nonage subgroup), sex, and year of hsCRP across key clinical subgroups: age <65 vs ≥65 years, BMI, Charlson comorbidity score, diabetes mellitus, and ejection fraction (EF ≥50 and EF <50). For BMI and the Charlson comorbidity score, we compared values below the median vs equal to or above the median. We evaluated multiplicative interaction between SI and the subgroup characteristic using interaction terms in the Cox models to identify whether HRs differed significantly across subgroups; additive interactions were not evaluated.

We used a threshold of *P* ≤ 0.05 for statistical significance. Data for this project are not publicly available, but code can be made available to Department of Veterans Affairs investigators upon request. The content is solely the responsibility of the authors and does not necessarily represent the official views of the VHA. This study was approved by the Stanford University Institutional Review Board and follows the Strengthening the Reporting of Observational Studies in Epidemiology reporting guidelines.

## Results

We identified 49,986 Veterans with HFpEF/HFmrEF with outpatient hsCRP test results. There were 8,696 (17.4%) with hsCRP <2 mg/L, 25,352 (50.7%) with hsCRP ≥2 mg/L and ≤10 mg/L, and 15,938 (31.9%) with hsCRP >10 mg/L (Figure 1). Among Veterans with hsCRP ≤10 mg/L, the median hsCRP level was 3.7 mg/L (IQR: 1.9-6.2 mg/L). The median follow-up was 3.4 years (IQR: 1.8-5.8). Among this cohort, the mean age was 71.0 years (SD: 9.6); 95.7% (32,576/34,048) were men. Veterans with SI (hsCRP ≥2 mg/L and ≤10 mg/L) were more likely to have atrial fibrillation (44.6% [11,306/25,352] vs 39.6% [3,442/8,696], *P* < 0.001), chronic kidney disease (29.2% [7,392/25,352] vs 23.6% [2,054/8,696], *P* < 0.001), and diabetes mellitus (54.8% [13,904/25,352] vs 46.8% [4,069/8,696], *P* < 0.001) compared to those without SI (hsCRP <2 mg/L). The mean ejection fraction was slightly higher among Veterans with SI (56.1% [SD: 8.4] vs 55.7% [SD: 8.5], *P* < 0.01) (Table 1).

**Figure 1:**
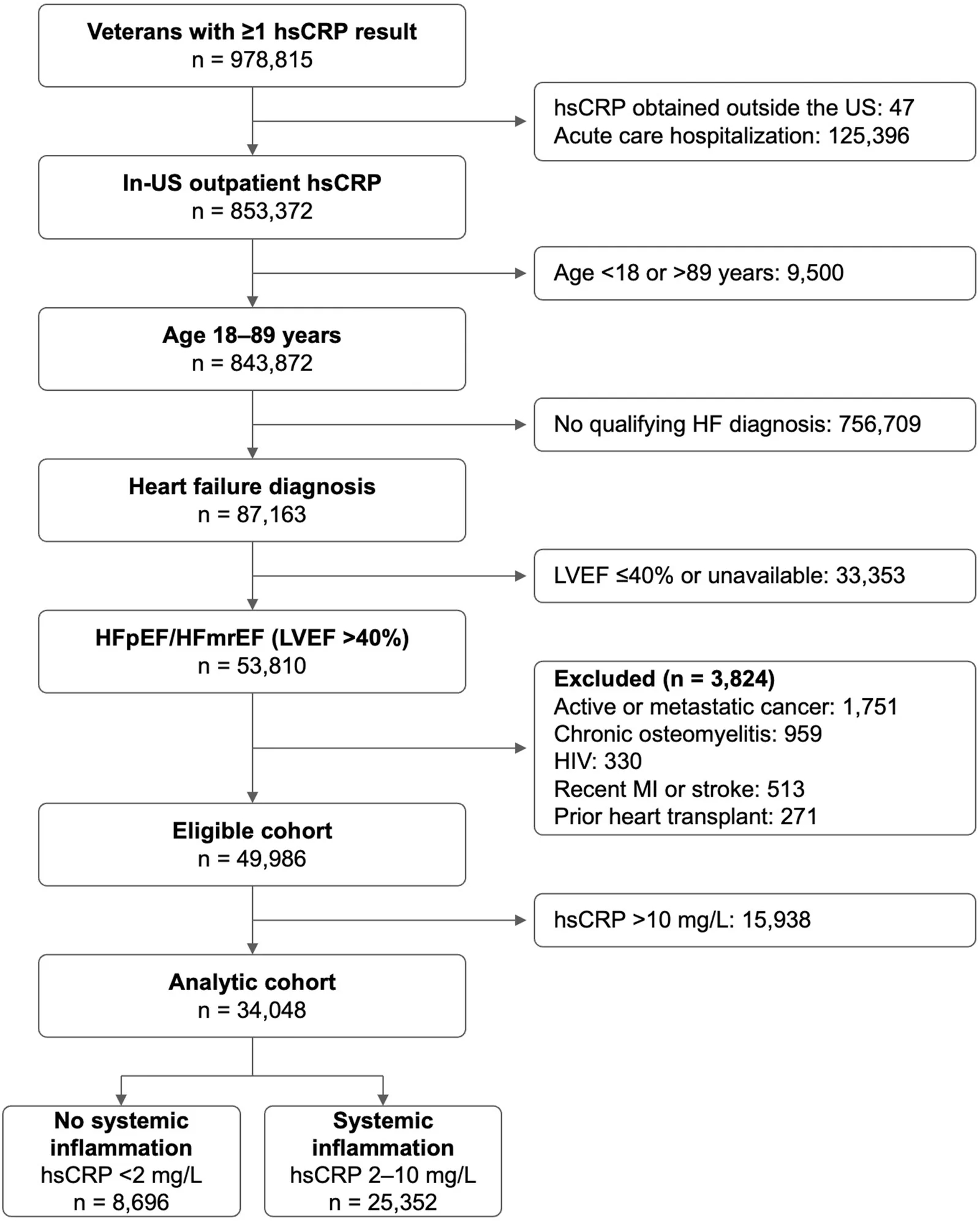
Study Flow Diagram Flow diagram of cohort identification, with the number of Veterans excluded at each step. HF = heart failure; HFmrEF = heart failure with mildly reduced ejection fraction; HFpEF = heart failure with preserved ejection fraction; hsCRP= high-sensitivity C-reactive protein; LVEF = left ventricular ejection fraction; MI = myocardial infarction.

**Table 1:** Baseline Characteristics of Patients With vs No Systemic Inflammation (SI)

	Overall (N = 34,048)	hsCRP >2 and <10 (n = 25,352)	hsCRP <2 (n = 8,696)	*P* Value
Age, y, mean (SD)	71.0 (9.58)	70.8 (9.50)	71.8 (9.77)	<0.001
Female, n (%)	1,472 (4.3)	1,091 (4.3)	381 (4.4)	0.781
Male, n (%)	32,576 (95.7)	24,261 (95.7)	8,315 (95.6)	
Race, n (%)				
Asian	259 (0.8)	157 (0.6)	102 (1.2)	<0.001
American Indian/Native American	359 (1.1)	262 (1.0)	97 (1.1)	
Black	6,089 (17.9)	4,480 (17.7)	1,609 (18.5)	
Pacific Islander/Native Hawaiian	323 (0.9)	236 (0.9)	87 (1.0)	
White	24,886 (73.1)	18,647 (73.6)	6,239 (71.7)	
Missing	2,132 (6.3)	1,570 (6.2)	562 (6.5)	
Ethnicity, n (%)				
Non-Hispanic/Latinx	30,249 (88.8)	22,540 (88.9)	7,709 (88.6)	0.495
Hispanic/Latinx	2,378 (7.0)	1,773 (7.0)	605 (7.0)	
Missing	1,421 (4.2)	1,039 (4.1)	382 (4.4)	
Rural designation, n (%)	7,116 (20.9)	5,343 (21.1)	1,773 (20.4)	0.047
Drive time to a primary care, minutes, median (IQR)	27.2 (26.1)	27.2 (27.4)	27.2 (21.7)	0.866
CDC SVI by quartile, n (%)				
<0.25	5,464 (16.0)	4,012 (15.8)	1,452 (16.7)	0.014
0.25-<0.50	7,630 (22.4)	5,600 (22.1)	2,030 (23.3)	
0.50-<0.75	8,499 (25.0)	6,374 (25.1)	2,125 (24.4)	
0.75-1.00	9,104 (26.7)	6,848 (27.0)	2,256 (25.9)	
Missing	3,351 (9.8)	2,518 (9.9)	833 (9.6)	
Smoking status				<0.001
Current	6,668 (19.6)	5,122 (20.2)	1,546 (17.8)	
Former	22,022 (64.7)	16,365 (64.6)	5,657 (65.1)	
Never	4,996 (14.7)	3,597 (14.2)	1,399 (16.1)	
Unknown	362 (1.1)	268 (1.1)	94 (1.1)	
Frailty				
Yes	12,609 (37.0)	9,778 (38.6)	2,831 (32.6)	<0.001
No	21,439 (63.0)	15,574 (61.4)	5,865 (67.4)	
Comorbidities, n (%)				
Cardiovascular				
Atherosclerotic cardiovascular disease	29,003 (85.2)	21,514 (84.9%)	7,489 (86.1%)	0.004
Atrial fibrillation/atrial flutter	14,748 (43.3)	11,306 (44.6)	3,442 (39.6)	<0.001
Cerebrovascular disease (ie, stroke and TIA)	9,708 (28.5)	7,156 (28.2)	2,552 (29.3)	0.047
Coronary artery disease	25,701 (75.5)	18,972 (74.8)	6,729 (77.4)	<0.001
Peripheral artery disease	14,647 (43.0)	11,228 (44.3)	3,419 (39.3)	<0.001
Myocardial infarction	2,588 (7.6)	1,833 (7.2)	755 (8.7)	<0.001
Valvular heart disease	11,645 (34.2)	8,537 (33.7)	3,108 (35.7)	<0.001
Ventricular arrhythmias	3,992 (11.7)	2,879 (11.4)	1,113 (12.8)	<0.001
Systemic autoimmune disorders				
Myositis	101 (0.3)	81 (0.3)	20 (0.2)	0.226
Multiple sclerosis	162 (0.5)	129 (0.5)	33 (0.4)	0.155
Rheumatoid arthritis	2,977 (8.7)	2,435 (9.6)	542 (6.2)	<0.001
Seronegative spondyloarthropathies	1,651 (4.8)	1,205 (4.8)	446 (5.1)	0.168
Systemic lupus erythematosus	359 (1.1)	282 (1.1)	77 (0.9)	0.084
Other systemic connective tissue disorders	309 (0.9)	247 (1.0)	62 (0.7)	0.031
Vasculitis	756 (2.2)	598 (2.4)	158 (1.8)	0.004
Other comorbidities				
Asthma	4,754 (14.0)	3,657 (14.4)	1,097 (12.6)	<0.001
Chronic kidney disease	9,446 (27.7)	7,392 (29.2)	2,054 (23.6)	<0.001
Chronic obstructive pulmonary disease	17,887 (52.5)	13,914 (54.9)	3,973 (45.7)	<0.001
Diabetes mellitus	17,973 (52.8)	13,904 (54.8)	4,069 (46.8)	<0.001
Hyperlipidemia	30,094 (88.4)	22,385 (88.3)	7,709 (88.6)	0.386
Hypertension	32,932 (96.7)	24,574 (96.9)	8,358 (96.1)	<0.001
Hypothyroidism	5,666 (16.6)	4,311 (17.0)	1,355 (15.6)	0.002
Liver disease				
Mild	4,222 (12.4)	3,175 (12.5)	1,047 (12.0)	0.245
Moderate or severe	1,170 (3.4)	937 (3.7)	233 (2.7)	<0.001
Charlson Comorbidity Index, median (IQR)	5 (3-8)	5 (3-8)	5 (3-7)	<0.001
1-2	5,128 (15.1)	3,457 (13.6)	1,671 (19.2)	<0.001
3-5	10,423 (30.6)	7,573 (29.9)	2,850 (32.8)	
>5	18,497 (54.3)	14,322 (56.5)	4,175 (48.0)	
Medication therapy, n (%)				
ACEI/ARB/ARNI	22,172 (65.1)	16,299 (64.3)	5,873 (67.5)	<0.001
ARNI	443 (1.3)	286 (1.1)	157 (1.8)	<0.001
Anticoagulants	11,225 (33.0)	8,638 (34.1)	2,587 (29.7)	<0.001
Antihyperlipidemic	25,766 (75.7)	18,822 (74.2)	6,944 (79.9)	<0.001
Statin				
High intensity	14,155 (41.6)	10,034 (39.6)	4,121 (47.4)	<0.001
Moderate intensity	10,287 (30.2)	7,788 (30.7)	2,499 (28.7)	
Low intensity	931 (2.7)	700 (2.8)	231 (2.7)	
No statin use	8,675 (25.5)	6,830 (26.9)	1,845 (21.2)	
Beta-blockers	25,020 (73.5)	18,601 (73.4)	6,419 (73.8)	0.426
Diuretics	22,925 (67.3)	17,644 (69.6)	5,281 (60.7)	<0.001
Glucagon-like peptide-1 receptor agonists (GLP-1RA)	951 (2.8)	715 (2.8)	236 (2.7)	0.63
Mineralocorticoid receptor antagonists	4,327 (12.7)	3,244 (12.8)	1,083 (12.5)	0.42
Sodium-glucose transport protein 2 inhibitors (SGLT-2i)	1,495 (4.4)	1,072 (4.2)	423 (4.9)	0.014
Physiologic measures, mean (SD)				
Body mass index, mean (SD)	31.85 (6.81)	32.44 (6.98)	30.13 (5.94)	<0.001
Missing, n (%)	362 (1.1)	267 (1.1)	95 (1.1)	0.804
Heart rate	73.8 (10.6)	74.3 (10.6)	72.3 (10.4)	<0.001
Missing, n (%)	129 (0.4)	89 (0.4)	40 (0.5)	0.185
Systolic blood pressure, mean (SD)	134.3 (13.9)	134.4 (13.9)	133.9 (13.9)	0.001
Missing, n (%)	127 (0.4)	88 (0.3)	39 (0.4)	0.216
Diastolic blood pressure, mean (SD)	74.2 (8.6)	74.2 (8.6)	74.4 (8.5)	0.013
Missing, n (%)	127 (0.4)	88 (0.3)	39 (0.4)	0.216
Ejection fraction (%)	55.9 (8.4)	56.1 (8.4)	55.7 (8.5)	<0.001
Missing, n (%)	4,607 (13.5)	3,324 (13.1)	1,283 (14.8)	<0.001
Laboratory values, mean (SD)				
Hemoglobin A1c (Hb A1c)	6.7 (1.4)	6.8 (1.4)	6.5 (1.3)	<0.001
Missing, n (%)	2,867 (8.4)	2,025 (8.0)	842 (9.7)	<0.001
eGFR (based on serum creatinine)	64.1 (24.9)	63.2 (25.4)	66.5 (23.3)	<0.001
Missing, n (%)	265 (0.8)	188 (0.7)	77 (0.9)	0.212
LDL-C	84.1 (30.1)	84.6 (30.2)	82.5 (29.7)	<0.001
<70 mg/L	12,706 (37.3)	9,262 (36.5)	3,444 (39.6)	<0.001
>70 mg/L	21,342 (62.7)	16,090 (63.5)	5,252 (60.4)	<0.001
Missing, n (%)	981 (2.9)	723 (2.9)		0.606
BNP, median (IQR)	148 (59-347)	151 (61-352)	136 (54-322)	<0.001
Missing, n (%)	15,317 (45.0%)	10,929 (43.1%)	4,388 (50.5%)	cent
NT-BNP, median (IQR)	684 (216-2,100)	740 (227-2,225)	529 (195-1,548)	<0.001
Missing, n (%)	29,079 (85.4%)	21,552 (85.0%)	7,527 (86.6%)	<0.001
Region, n (%)				
Northeast	4,321 (12.7)	3,207 (12.6)	1,114 (12.8)	0.557
Midwest	3,962 (11.6)	2,991 (11.8)	971 (11.2)	
South	13,485 (39.6)	10,033 (39.6)	3,452 (39.7)	
West	11,493 (33.8)	8,544 (33.7)	2,949 (33.9)	
Non-continental United States^a^	787 (2.3)	577 (2.3)	210 (2.4)	

ACEI/ARB/ARNI = angiotensin-converting enzyme inhibitor/angiotensin receptor blocker/angiotensin receptor neprilysin inhibitor; CDC = Centers for Disease Control; eGFR = estimated glomerular filtration rate; hsCRP= high-sensitivity C-reactive protein. Physiologic Measures: include most recent; LDL-C = low-density lipoprotein cholesterol; NT-BNP = N-terminal brain natriuretic peptide; SVI = Social Vulnerability Index; TAIA = transient ischemic attack.

a Alaska, Hawaii, Puerto Rico, U.S. Virgin Islands.

Veterans with SI were less likely to be on lipid-lowering therapy (74.2% [18,822/25,352] vs 79.9% [6,944/8,696], *P* < 0.001) and angiotensin-converting enzyme inhibitor/angiotensin receptor blocker therapy (64.3% [16,299/25,352] vs 67.5% [5,873/8,696], *P* < 0.001), whereas rates of anticoagulation were higher in Veterans with SI than those without SI (34.1% [8,638/25,352] vs 29.7% [2,587/8,696], *P* < 0.001). Across both groups, glucagon-like peptide-1 receptor agonist treatment (2.8% [715/25,352] vs 2.7% [236/8,696], *P* = 0.63) and sodium-glucose transport protein 2 inhibitor treatment (4.2% [1,072/25,352] vs 4.9% [423/8,696], *P* = 0.014) were low (Table 1).

### Clinical outcomes

The primary 3-component MACE outcome (the composite incidence of death, HF hospitalization, and ED HF visits) rate was 15.1/100 patient-years (95% CI: 14.9-15.4) among those with SI vs 11.8/100 patient-years (95% CI: 11.4-12.1) among those without SI (Figure 2, Central Illustration). After adjustment for clinical characteristics, SI was associated with a higher hazard of the primary outcome (HR: 1.28; 95% CI: 1.24-1.34). When hsCRP was modeled as quartiles, we found a stepwise association between higher levels of hsCRP and the MACE outcome (Supplemental Table 2). Veterans with hsCRP in the highest quartile (hsCRP >6.2 mg/L and <10 mg/L) had a 43% higher hazard of the primary outcome (HR: 1.43; 95% CI: 1.37-1.50) compared with Veterans with hsCRP in the lowest quartile (hsCRP <1.905 mg/L). As a continuous variable, every 1 SD of the hsCRP level was associated with a 28% higher hazard of the MACE outcome (HR: 1.28; 95% CI: 1.24-1.33). When modeled as a spline, there was an approximately linear association between hsCRP and the outcome (Supplemental Figure 1).

**Figure 2:**
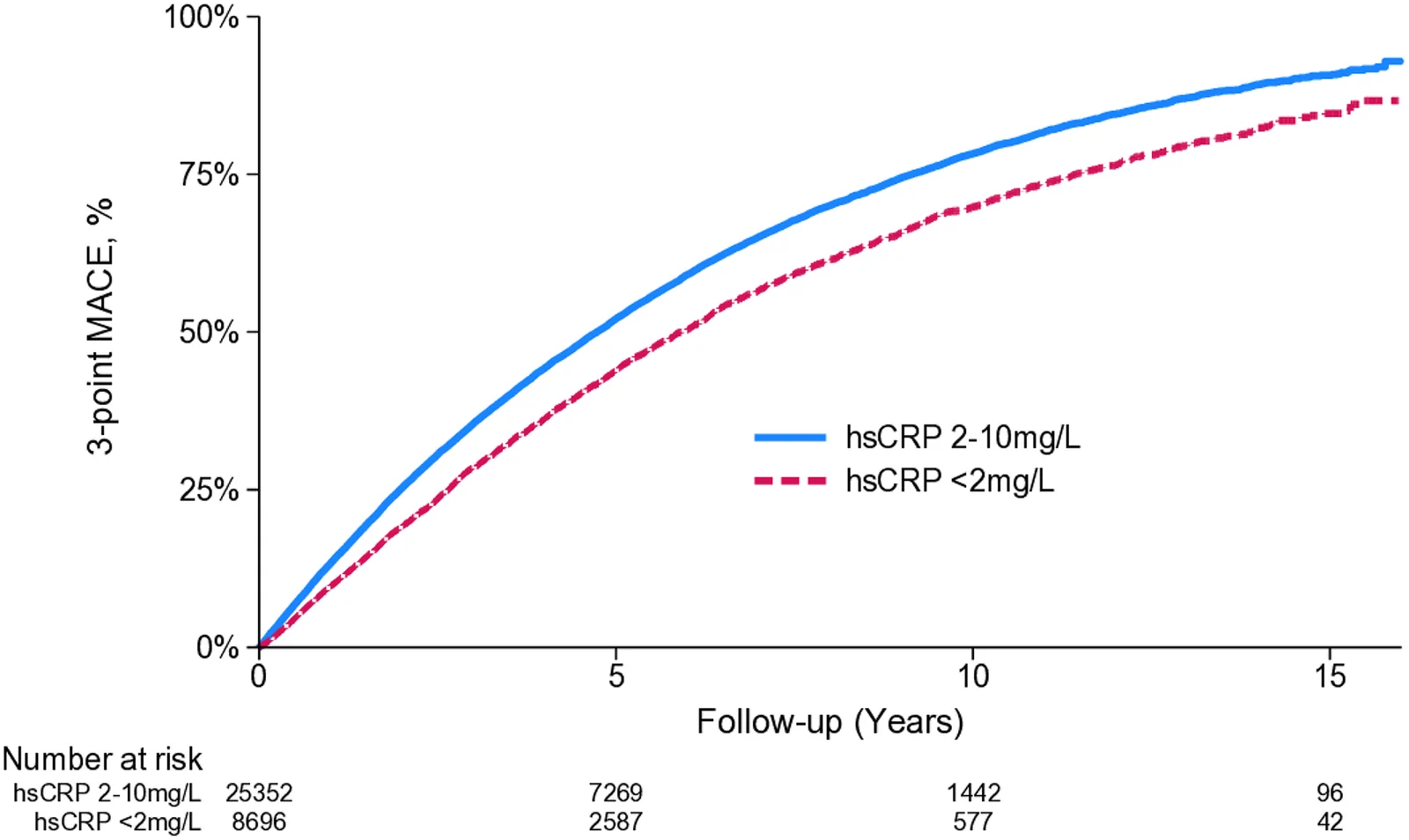
Association Between Systemic Inflammation and Composite Major Adverse Cardiovascular Event Outcome Cumulative incidence curves of the major adverse cardiovascular event outcome for patients with (blue solid line) and without (red dotted line) systemic inflammation. The number of participants at risk in each group is shown below the x-axis at each evaluated time point. The log-rank *P* value comparing groups is displayed within the graph. MACE = major adverse cardiovascular events; other abbreviation as in Figure 1.

**Central Illustration:**
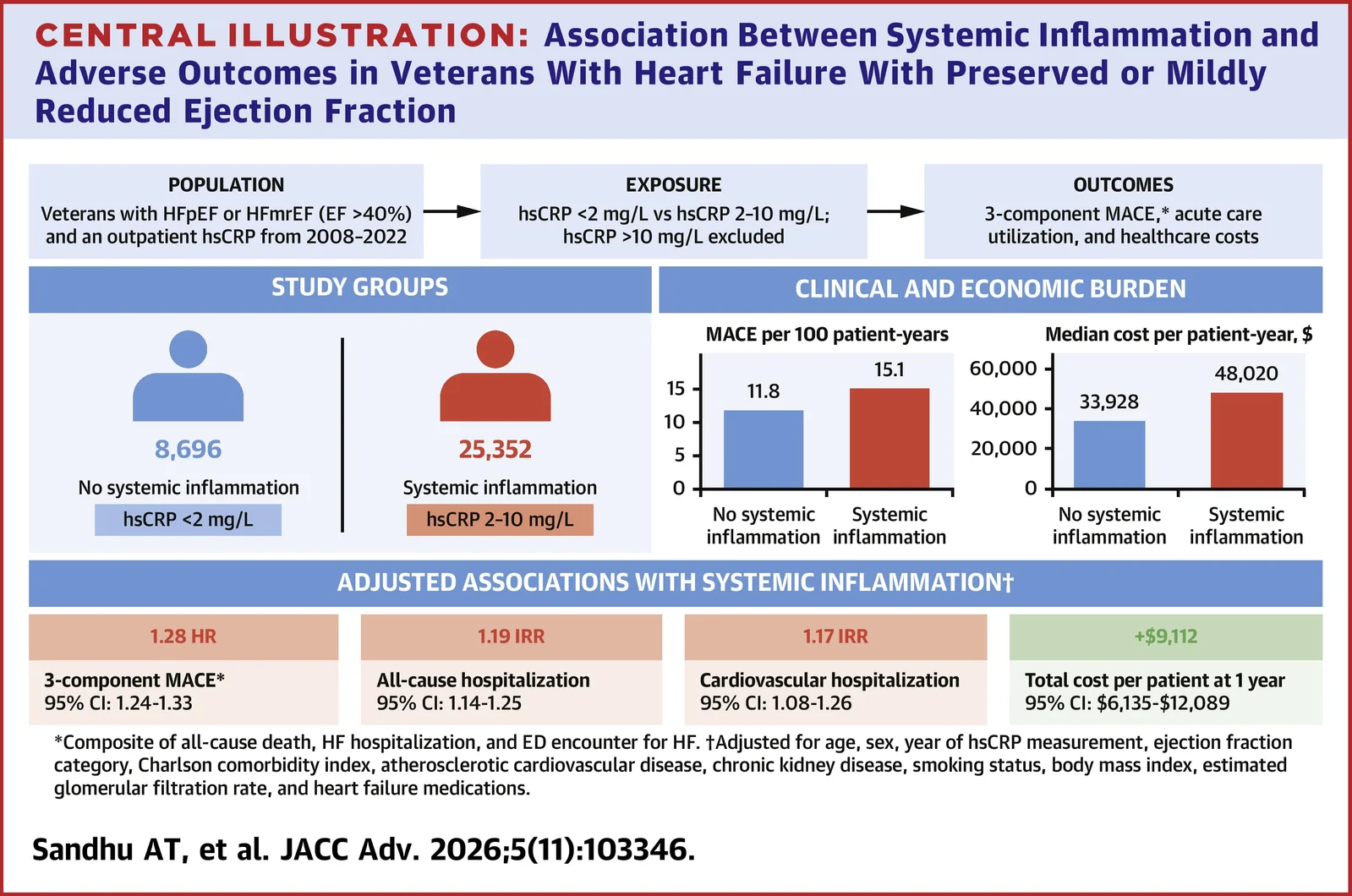
Association Between Systemic Inflammation and Adverse Outcomes in Veterans With Heart Failure With Preserved or Mildly Reduced Ejection Fraction ED = emergency department; IRR = incidence rate ratio; other abbreviations as in Figure 1, Figure 2, Figure 3.

There was a significant association between SI and each of the individual components of the composite MACE outcome (Table 2). In the adjusted model, SI was associated with increased hazard of all-cause death (HR: 1.29; 95% CI: 1.25-1.34) and cardiovascular death (HR: 1.23; 95% CI: 1.16-1.31) (Table 2). For each secondary outcome, there was a stepwise association between higher hsCRP quartiles and increased risk of outcomes (Supplemental Table 2). SI was also associated with the composite outcome of death, HF hospitalization, ED visit for HF, myocardial infarction, or stroke (HR: 1.26; 95% CI: 1.22-1.30).

**Table 2:** Clinical Outcome Rates (Event Rates per 100 Patient-Years) of Patients With vs Without Systemic Inflammation

	Overall (N = 34,048)	With SI (hsCRP ≥2 and ≤ 10) (n = 25,352)	Without SI (hsCRP <2) (n = 8,696)	*P* Value^e^	HR With SI	
					Model A^a^	Model B^b^
3-point MACE^c^	14.25 (14.04-14.44)	15.13 (14.89-15.37)	11.78 (11.42-12.13)	<0.0001	1.37^d^ (1.32-1.42)	1.28^d^ (1.24-1.34)
All-cause death	11.88 (11.7-12.06)	12.59 (12.37-12.8)	9.88 (9.56-10.19)	<0.0001	1.37^d^ (1.32-1.42)	1.29^d^ (1.25-1.34)
Cardiovascular death	4.75 (4.62-4.86)	4.96 (4.82-5.1)	4.11 (3.89-4.33)	<0.0001	1.31^d^ (1.23-1.39)	1.23^d^ (1.16-1.31)
Heart failure hospitalization	2.56 (2.47-2.64)	2.74 (2.63-2.83)	2.05 (1.91-2.2)	<0.0001	1.41^d^ (1.29-1.53)	1.24^d^ (1.14-1.35)
ED visit for heart failure	1.75 (1.67-1.81)	1.85 (1.77-1.93)	1.45 (1.33-1.57)	<0.0001	1.33^d^ (1.20-1.47)	1.22^d^ (1.10-1.35)

ED = emergency department; MACE = major adverse cardiovascular events; MI = myocardial infarction; SI = systemic inflammation; GLP-1 RA = glucagon-like peptide-1 receptor agonists; other abbreviation as in Table 1.

a Model A adjusted for age, sex, and year of hsCRP measurement.

b Model B adjusted for age, sex, year of hsCRP measurement, Charlson Comorbidity Index, prior MI, prior coronary revascularization, smoking status, body mass index, GFR, lipid-lowering therapies, antiplatelets, anticoagulants, angiotensin-converting enzyme inhibitors/angiotensin receptor blockers, SGLT-2i, and GLP-1 RA.

c 3-point MACE includes all-cause death, heart failure hospitalization, and ED visit for heart failure.

d Indicates *P* < 0.05 for HRs evaluating association between SI (hsCRP ≥2 mg/L and ≤10 mg/L) and the outcome.

e The *P* value for the difference in incidence rates between Veterans with SI and without SI.

We tested the interaction between SI and patient characteristics in subgroup analyses. SI was significantly associated with the MACE outcome, all-cause death, and cardiovascular death in each subgroup of interest (Figure 3). There was a significant interaction between diabetes mellitus and SI. The association between SI and MACE was stronger among Veterans without diabetes (HR: 1.38; 95% CI: 1.31-1.45) compared with those with diabetes (HR: 1.29; 95% CI: 1.23-1.35) (*P* = 0.04 for interaction).

**Figure 3:**
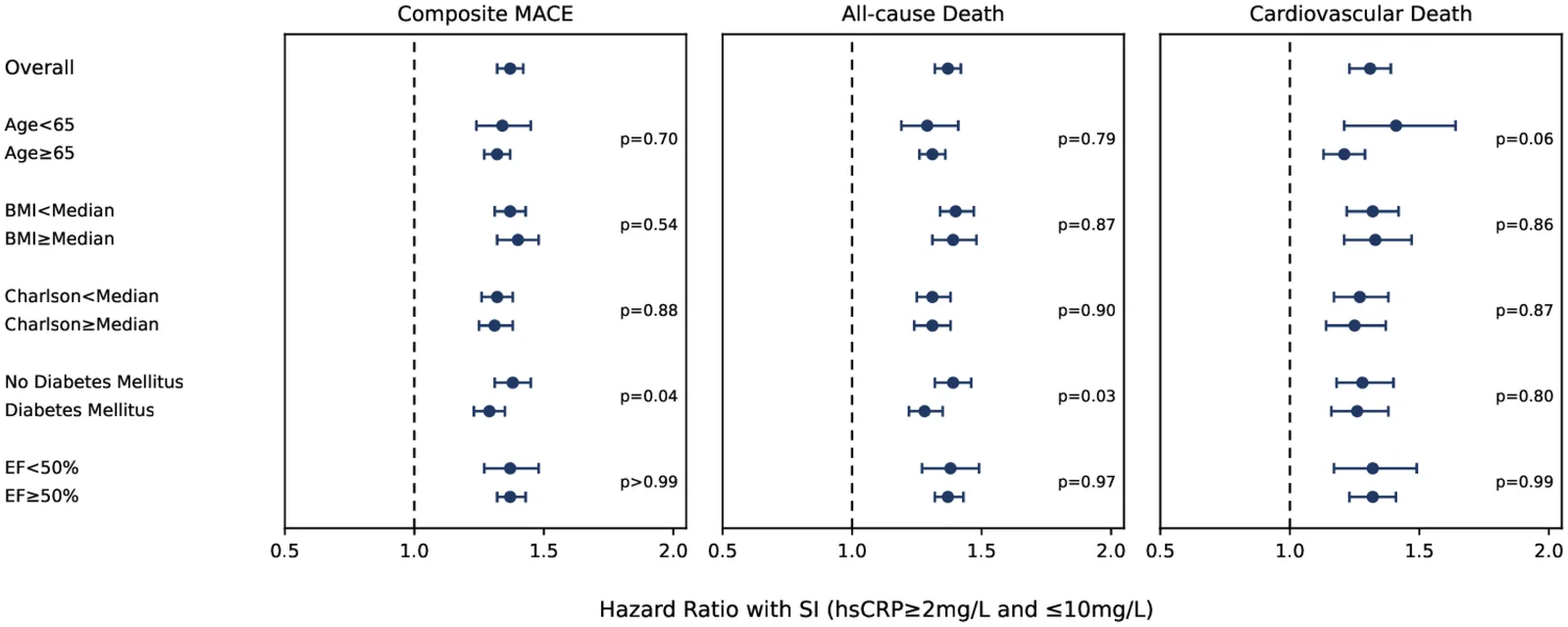
Adjusted Association Between Systemic Inflammation and Outcomes Stratified by Veteran Characteristics Subgroup analysis of the association between systemic inflammation and outcomes. *P* values for multiplicative interaction between systemic inflammation and each subgroup characteristic are reported for all subgroups. BMI = body mass index; EF = ejection fraction; SI = systemic inflammation; other abbreviations as in Figures 1 and 2.

We completed multiple sensitivity analyses. First, after excluding the 5,523 Veterans with systemic autoimmune conditions, we found the rate of MACE remained significantly higher among the 21,004 Veterans with SI (15.12 per 100 patient-years; 95% CI: 14.86-15.39) compared with the 7,521 Veterans without SI (11.73 per 100 patient-years; 95% CI: 11.35-12.11) (*P* < 0.001). The results were similar for each of the other clinical endpoints (Supplemental Table 3). Second, after restricting to the first hsCRP test among 28,051 Veterans, we found the MACE rate remained higher among Veterans with SI (14.69 per 100 patient-years; 95% CI: 14.43-14.96) compared with Veterans without SI (11.55 per 100 patient-years; 95% CI: 11.19-11.92) (*P* < 0.001). Third, including Veterans with hsCRP >10 mg/L led to higher cardiovascular event rates among Veterans with hsCRP >2 mg/L (Supplemental Table 4). Fourth, the association between SI and MACE was similar after excluding Veterans with missing EF data (HR: 1.28; 95% CI: 1.24-1.33) and after excluding those with improved EF (HR: 1.28; 95% CI: 1.23-1.32). Fifth, a complete case analysis restricted to Veterans with no missing data (n = 31,836) yielded a similar association between SI and MACE (HR: 1.28; 95% CI: 1.23-1.32) (Supplemental Table 5). Finally, after excluding the 383 individuals with antimicrobial medication fill within 30 days prior or 14 days after hsCRP measurement, we found SI remained associated with MACE (HR: 1.28; 95% CI: 1.24-1.33).

### Acute care utilization and health care cost

We found higher acute care utilization among Veterans with SI (Supplemental Table 6). Among Veterans with 1 year of follow-up, SI was associated with a 19% increase in the rate of all-cause hospitalizations after adjustment for clinical characteristics (IRR: 1.19; 95% CI: 1.14-1.25). SI was associated with a 17% increase in the rate of cardiovascular hospitalizations (IRR: 1.17; 95% CI: 1.08-1.26). The associations remained significant across shorter and longer follow-up durations (Table 3). There was not a significant association between SI and HF hospitalizations after adjustment to clinical characteristics. Finally, the frequency of ED encounters was greater among those with SI (Table 3).

**Table 3:** Association Between SI and Acute Care Utilization Rate

Outcomes	Follow-Up Period							
	6 Months (n = 32,594)^a^		1 Year (n = 30,935)^a^		3 Years (n = 19,069)^a^		5 Years (n = 11,074)^a^	
	Model A^b^	Model B^c^	Model A^b^	Model B^c^	Model A^b^	Model B^c^	Model A^b^	Model B^c^
All-cause hospitalization	1.31^d^ (1.24-1.38)	1.22^d^ (1.16-1.29)	1.28^d^ (1.23-1.35)	1.19^d^ (1.14-1.25)	1.29^d^ (1.23-1.35)	1.19^d^ (1.13-1.24)	1.26^d^ (1.19-1.33)	1.16^d^ (1.09-1.22)
Cardiovascular hospitalization	1.32^d^ (1.20-1.45)	1.23^d^ (1.12-1.35)	1.25^d^ (1.16-1.35)	1.17^d^ (1.08-1.26)	1.21^d^ (1.12-1.30)	1.11^d^ (1.03-1.19)	1.21^d^ (1.11-1.31)	1.09^d^ (1.00-1.18)
Heart failure hospitalization	1.36^d^ (1.04-1.78)	1.11 (0.84-1.46)	1.24^d^ (1.00-1.54)	1.09 (0.88-1.36)	1.33^d^ (1.10-1.60)	1.14 (0.94-1.37)	1.36^d^ (1.09-1.69)	1.18 (0.94-1.47)
HF emergency department	1.72^d^ (1.22-2.42)	1.62^d^ (1.14-2.30)	1.40^d^ (1.10-1.79)	1.31^d^ (1.02-1.67)	1.53^d^ (1.24-1.89)	1.36^d^ (1.10-1.68)	1.26^d^ (1.01-1.57)	1.12 (0.89-1.41)
Emergency department	1.16^d^ (1.12-1.20)	1.13^d^ (1.09-1.17)	1.15^d^ (1.11-1.19)	1.13^d^ (1.09-1.17)	1.18^d^ (1.14-1.23)	1.14^d^ (1.10-1.18)	1.19^d^ (1.13-1.24)	1.14^d^ (1.09-1.19)

HF = heart failure; other abbreviations as in Tables 1 and 2.

a Each analysis excludes individuals with death or loss to follow-up before the end of the follow-up period.

b Model A adjusted for age, sex, and year of hsCRP measurement.

c Model B adjusted for age, sex, year of hsCRP measurement, Charlson Comorbidity Index, prior MI, prior coronary revascularization, smoking status, body mass index, GFR, lipid-lowering therapies, antiplatelets, anticoagulants, angiotensin-converting enzyme inhibitors/angiotensin receptor blockers, SGLT-2i, and GLP-1 RA.

d Indicates *P* < 0.05 for incidence rate ratio between SI (hsCRP ≥2 mg/L and ≤10 mg/L) and the outcome.

The median cost per patient-year was $48,020 (IQR: $21,990-$98,921) among those with SI and $33,928 (IQR: $15,171-$71,484) among those without SI (*P* < 0.001). Inpatient, outpatient, pharmacy, and cardiovascular-related costs were all higher among those with SI (Table 4).

**Table 4:** Cost of Care Stratified by SI vs No SI (2023 USD)

Per Patient-Year of Follow-Up	Overall (N = 34,048)	With SI (hsCRP ≥2 and ≤ 10) (n = 8,696)	Without SI (hsCRP <2) (n = 25,352)	*P* Value^a^
Total	43,994 (19,833-91,378)	48,020 (21,990-98,921)	33,928 (15,171-71,484)	<0.001
Inpatient	14,550 (1,091-46,549)	16,900 (2,020-51,691)	8,889 (0-32,819)	<0.001
Cardiovascular	6,213 (1,365-19,920)	6,850 (1,534-21,351)	4,580 (1,061-15,661)	<0.001
Heart failure	353 (0-4,193)	449 (0-4,948)	168 (0-2,377)	<0.001
Outpatient	18,757 (9,657-34,481)	19,834 (10,284-36,218)	15,887 (8,204-29,577)	<0.001
Pharmacy	2,104 (983-4,426)	2,226 (1,050-4,620)	1,788 (791-3,842)	<0.001

Values are median (IQR). USD = United States dollars; other abbreviations as in Tables 1 and 2.

a The *P* value for the difference in costs between Veterans with SI and without SI.

Over 1 year of follow-up, SI was associated with $9,112 (95% CI: $6,135-$12,089) in additional total costs and $5,648 (95% CI: $4,064-$7,231) in inpatient costs after adjustment for clinical characteristics. Costs were also significantly greater for cardiovascular-related, outpatient, and pharmacy costs (Table 5). The increase in costs associated with SI increased over time (Table 5). At 5 years of follow-up, SI was associated with $28,151 (95% CI: $19,302-37,001) in additional costs per patient. The association between SI and costs was similar with a Tobit model (Supplemental Table 7).

**Table 5:** Marginal Cost of SI Based on Follow-Up Duration and Cost Category (USD 2023)^a^^,^^b^

Outcomes	6-Month Follow-Up		1-Year Follow-Up		3-Year Follow-Up		5-Year Follow-Up	
	Model A^c^	Model B^d^	Model A^c^	Model B^d^	Model A^c^	Model B^d^	Model A^c^	Model B^d^
Total	8,267^a^ (4,004-12,530)	4,810^a^ (2,475-7,145)	14,489^a^ (9,293-19,684)	9,112^a^ (6,135-12,089)	36,087^a^ (24,394-47,779)	22,456^a^ (15,039-29,873)	47,671^a^ (38,011-57,332)	28,151^a^ (19,302-37,001)
Inpatient	3,987^a^ (2,856-5,118)	2,685^a^ (1,571-3,799)	7,852^a^ (6,259-9,445)	5,648^a^ (4,064-7,231)	17,449^a^ (13,926-20,972)	11,654^a^ (7,957-15,350)	27,358^a^ (21,598-33,117)	19,347^a^ (13,616-25,077)
Cardiovascular^e^	1,385^a^ (753.2-2,017)	947.8^a^ (269.6-1,626)	2,527^a^ (1,581-3,474)	1,624^a^ (572.2-2,676)	5,096^a^ (2,752-7,441)	3,143^a^ (697.9-5,588)	7,948^a^ (4,651-11,245)	4,708^a^ (1,225-8,191)
Outpatient	2,213^a^ (1,717-2,709)	1,204^a^ (729.3-1,679)	3,907^a^ (3,044-4,771)	2,092^a^ (1,288-2,896)	11,988^a^ (9,273-14,703)	7,308^a^ (5,013-9,604)	17,660^a^ (12,116-23,203)	7,076^a^ (2,716-11,437)
Heart failure^f^	606.4^a^ (349.8-863.1)	427.9^a^ (160.5-695.3)	1,553^a^ (1,212-1,895)	1,208^a^ (842.9-1,574)	3,285^a^ (1,942-4,628)	2,454^a^ (1,196-3,712)	4,818^a^ (3,433-6,202)	2,906^a^ (1,423-4,389)
Pharmacy	924.9 (−240.0 to 2,090)	407.5 (−180.0 to 995.0)	1,591 (−221.4 to 3,403)	710.2 (−279.4 to 1,700)	4,715 (−358.9 to 9,789)	2,385 (−397.2 to 5,167)	3,259^a^ (1,303-5,215)	2,288^a^ (457.4-4,118)

ICD = International Classification of Diseases; other abbreviations as in Tables 1, 2, and 4.

a Indicates *P* < 0.05 for adjusted marginal cost difference between Veterans with SI (hsCRP ≥2 mg/L and ≤10 mg/L) and without SI (hsCRP <2 mg/L).

b Each analysis excludes individuals with death or loss to follow-up before the end of the follow-up period.

c Model A adjusted for age, sex, and year of hsCRP measurement.

d Model B adjusted for age, sex, year of hsCRP measurement, Charlson Comorbidity Index, prior MI, prior coronary revascularization, smoking status, body mass index, GFR, lipid-lowering therapies, antiplatelets, anticoagulants, angiotensin-converting enzyme inhibitors/angiotensin receptor blockers, SGLT-2i, and GLP-1 RA.

e Cardiovascular-related costs were identified based on the presence of associated ICD codes indicative of cardiovascular disease.

f Heart failure costs were based on the presence of associated ICD codes indicative of heart failure.

## Discussion

In a national cohort of Veterans with HFpEF/HFmrEF, we found that SI identified during clinical practice was associated with increased hazard of all-cause death, HF hospitalizations, and ED visits for HF. Furthermore, SI was associated with increased acute care utilization and health care costs. These results demonstrate a substantial disease burden associated with SI in HFpEF/HFmrEF and support the relationship between SI and HFpEF/HFmrEF risk.

There is a growing interest in the inflammation hypothesis for the pathogenesis of HFpEF/HFmrEF.^6^^,^^7^ Chronic kidney disease, diabetes mellitus, hypertension, and obesity are each associated with increased SI.^8^ SI causes myocardial oxidative stress and impairs endothelial dysfunction, which is thought to cause myocardial hypertrophy and fibrosis, impaired relaxation, and HFpEF.^6^^,^^7^ The relationship between SI and HFpEF has previously been illustrated in both animal and human models.^35^^,^^36^

Multiple clinical trials have found over 50% of patients with HFpEF/HFmrEF have SI based on hsCRP measurement.^11^^,^^37^, ^38^, ^39^ In the POSEIDON (Prevalence of Systemic Inflammation in Patients with Atherosclerotic Cardiovascular Disease and/or Heart Failure Study) study, which prospectively measured hsCRP in 11,809 patients with HF across 18 countries, elevated hsCRP (≥2 mg/L) was present in approximately 38% of patients. Patients with elevated hsCRP were more likely to be female and to have chronic kidney disease, obesity, worse NYHA class, and higher natriuretic peptide levels, consistent with our findings in the VA.^40^ There is also prior evidence that the presence of SI is associated with increased risk. Ferreira et al^11^ found those with SI had over 2-fold higher rates of cardiovascular death and HF hospitalizations compared with those without SI in a subset of 232 patients in the TOPCAT (Aldosterone Antagonist Therapy for Adults With Heart Failure and Preserved Systolic Function) trial. Koller et al^12^ demonstrated that a one standard deviation increase in the CRP level was associated with a 20% increase in the hazard of death among 459 individuals with HFpEF. Our analysis extends prior prospective cohorts and trial substudies, which were generally limited to several 100 patients, to a large, nationwide population identified during routine clinical care. In addition to confirming a graded association between hsCRP and adverse cardiovascular outcomes after multivariable adjustment, we demonstrate for the first time in HFpEF/HFmrEF that SI is associated with substantially higher acute care utilization and health care costs, underscoring the broader clinical and economic burden of this phenotype.

HFpEF/HFmrEF is a highly heterogeneous condition in terms of etiology and disease course. However, existing risk scores only demonstrate modest discrimination.^41^ Given hsCRP is a significant predictor of adverse outcomes after adjustment for other clinical characteristics, it may be a valuable characteristic for future risk prediction among individuals with HFpEF/HFmrEF. Overall, our cohort had similar or higher rates of all-cause and cardiovascular mortality as prior outpatient registries.^42^, ^43^, ^44^ We consistently found those with SI have higher rates of all-cause mortality, cardiovascular mortality, and HF hospitalization as those without SI.

Heart failure is responsible for over $30 billion in health care costs with an anticipated increase to over $120 billion by 2050.^45^ Among Veterans with HFmrEF/HFpEF, we found per-patient total costs exceeded $40,000 per year. In comparison, Bhatnagar and colleagues estimated a mean annual per-patient expenditure of $28,950 for individuals with HF using the Medical Expenditure Panel Survey.^46^ In our analysis, Veterans with SI had higher annual inpatient, outpatient, and pharmaceutical costs. Of note, the largest absolute difference was among inpatient costs, consistent with the increased frequency of acute care hospitalizations for Veterans with SI observed in our analysis. Because the indication for hsCRP testing could not be determined, the higher costs observed among patients with SI should be interpreted as associations rather than as costs caused by inflammation. The clinical circumstances prompting testing may themselves be related to subsequent health care utilization and cost, although patients in both arms underwent hsCRP testing.

Our findings have several implications for clinical practice. HsCRP is a widely available biomarker, and our results indicate that an elevated outpatient hsCRP identifies a subgroup of patients with HFpEF/HFmrEF at substantially higher risk for death, HF hospitalization, and acute care utilization. Because existing HFpEF/HFmrEF risk scores offer only modest discrimination, measuring hsCRP at a stable, ambulatory visit (without recent acute infection) may be a practical way to refine prognostic assessment and to prioritize closer follow-up, optimization of guideline-directed therapy, and goals-of-care discussions. However, there is not currently evidence that hsCRP-guided escalation of therapy improves outcomes or that hsCRP should be used as a treatment target outside of a trial. Two recent therapies that have demonstrated benefit among patients with HFpEF/HFmrEF—glucagon-like peptide-1 receptor agonists and sodium-glucose transport protein 2 inhibitors—both indirectly decrease SI.^38^^,^^47^ However, with both drug classes, the anti-inflammatory effect is moderate, and the majority of individuals with SI continue to have elevated hsCRP.^48^^,^^49^

It remains unclear if directly treating SI will improve outcomes among individuals with HFpEF/HFmrEF. After tumor necrosis factor-alpha was found to potentially play a role in HFrEF progression, 2 prior trials failed to find a benefit with tumor necrosis factor-alpha inhibitors.^50^^,^^51^ However, the pathophysiology of HFrEF is distinct from that of HFpEF/HFmrEF. Furthermore, in the Canakinumab Anti-Inflammatory Thrombosis Outcomes Study, canakinumab (a monoclonal antibody targeting interleukin-1) led to a significant decrease in HF hospitalizations.^52^ Because interleukin-6 drives hepatic CRP production, an ongoing trial is evaluating whether the anti-IL-6 antibody ziltivekimab improves outcomes among individuals with HFpEF/HFmrEF and hsCRP-defined SI. This trial will help define whether SI can be directly targeted with anti-inflammatory therapies.

### Studyt limitations

There are multiple important limitations to our analysis. First, the indication for outpatient hsCRP testing could not be determined and may differ from a general HFpEF/HFmrEF population, which could affect generalizability. Our cohort had substantially higher rates of elevated hsCRP than the POSEIDON cohort, in which hsCRP was systematically measured at routine visits.^40^ However, the clinical correlates of elevated hsCRP were similar to those in POSEIDON. Additionally, all patients in both groups underwent the same outpatient testing, supporting the internal validity of the comparison. Second, there may be confounding of the association between hsCRP levels and cardiovascular outcomes, as this is an observational analysis. There may be additional unmeasured comorbidities that were associated with hsCRP level and our outcomes. Third, we used a hierarchical selection of hsCRP levels among Veterans with multiple measurements. However, our results were similar when restricting to the first hsCRP measurement. Finally, our cohort was disproportionately male; this limited our ability to evaluate the association between hsCRP and outcomes among women.

SI is associated with significantly increased cardiovascular events and health care costs among Veterans with HFpEF/HFmrEF. In a cohort that has substantial morbidity, the presence of SI may be an important marker for identifying those at highest risk. Identifying strategies to improve outcomes in this high-risk and high-cost subgroup of patients with HFpEF/HFmrEF will be important.

## Funding support and author disclosures

This work was funded by Novo Nordisk. This material is the result of work supported with resources and the use of facilities at the VA Palo Alto Healthcare System, Palo Alto, CA. Support for the Department of Veterans Affairs (VA) and Centers for Medicare and Medicaid Services data was provided by the VA and the VA Information Resource Center. The funders contributed to the design of the study, interpretation of the data, and review of the manuscript. The funders had no role in the collection, management, analysis, or the decision to submit the manuscript for publication. Dr Sandhu has received research funding outside the submitted work from NIH, the American Heart Association, Amgen, AstraZeneca, Bayer, and Novartis; and consulting fees from Cleerly, Holosis Health, and Reprieve Cardiovascular. Dr Varshney has received consulting fees from Broadview Ventures, Hippocratic AI, ECHAS, and Baim Clinical Research Institute. Drs Gromovsky, Khachatourian, Skaar, Sandhu, and Tonnu-Mihara were employees of Novo Nordisk during the study. Drs Gromovsky, Khachatourian, and Skaar have NNI stock. Dr Skaar has equity in Trinity Life Sciences. All other authors have reported that they have no relationships relevant to the contents of this paper to disclose.

## Appendix

For an expanded Methods section, supplemental tables and figures, please see the online version of this paper.
