# Supplementary material for: Systemic Inflammation and Adverse Heart Failure Outcomes: Insights From the Veterans Affairs Health Care System
Source: JACC Adv. 2026 Oct 23;5(11):103346. doi: 10.1016/j.jacadv.2026.103346 (PMC13721086; doi:10.1016/j.jacadv.2026.103346)
Supplement: Supplementary file 1 [file mmc1.docx]

**Supplement**

Table of Contents

[Supplement Methods	2](#_Toc238920676)

[Supplement Table 1. ICD Code List for Comorbidities, Laboratory Tests, and Procedures	3](#_Toc238920677)

[Supplement Table 2. Association Between hsCRP Quartiles and Adverse Cardiovascular Outcomes	8](#_Toc238920678)

[Supplement Table 3. Rates of Adverse Cardiovascular Outcomes among Veterans with and without SI Excluding Individuals with Systemic Autoimmune Conditions	9](#_Toc238920679)

[Supplement Table 4. Rates of Adverse Cardiovascular Outcomes with and without SI Including hsCRP>10mg/L	10](#_Toc238920680)

[Supplement Table 5: Hazard Ratio of Systemic Inflammation (hsCRP>2) In Primary Analysis and Complete Case Analysis of patients with vs. without Systemic Inflammation*	11](#_Toc238920681)

[Supplement Table 6. Annual Rates of Acute Care Utilization among Veterans with and without SI*	12](#_Toc238920682)

[Supplement Table 7. Marginal Cost of SI among Veterans with and without SI Using Tobit Model*	13](#_Toc238920683)

[Supplement Figure 1. Association Between hsCRP Modeled as a Spline and Composite MACE Outcome	14](#_Toc238920684)

**Supplement Methods**

Among the covariates included in the fully adjusted model (Model B), only body mass index and estimated glomerular filtration rate had missing values, missing in 5.8% (1,968/34,048) and 0.8% (265/34,048) of Veterans, respectively. All other model covariates were complete. Ejection fraction was non-missing by construction: when unavailable from the natural language processing algorithm, HFpEF/HFmrEF was identified using previously validated diagnosis codes for diastolic HF. We classified these as preserved ejection fraction (HFpEF) given we previously found 79% of individuals with diastolic HF ICD-10 code (I50.3X) had EF≥50% compared with 9% with EF 40-50%.^1^ The influence of this classification was assessed in a separate sensitivity analysis restricted to Veterans with a measured ejection fraction.

Given missingness below 6% for both covariates bounds the influence of any imputation approach on the effect estimates, and we therefore used mean imputation with missing indicators. We did fit logistic regression models predicting missingness for each covariate from the remaining model covariates; observed characteristics predicted missingness poorly (pseudo-R² = 0.03 and 0.15), indicating that conditional imputation would yield values close to the marginal mean and that multiple imputation would be expected to produce similar estimates. We assessed this empirically with two complete case analyses ([1] excluding those with missing BMI or eGFR and [2] also excluding those with imputed EF). The results were very similar with these sensitivity analyses. (Supplement Table 5).

# **Supplement Table 1. ICD Code List for Comorbidities, Laboratory Tests, and Procedures**

| **Condition** | **ICD- code List** |
| --- | --- |
| Diabetes Mellitus | 24900; 24901; 24910; 24911; 24920; 24921; 24930; 24931; 24940; 24941; 24950; 24951; 24960; 24961; 24970; 24971; 24980; 24981; 24990; 24991; 25002; 25003; 25010; 25012; 25013; 25021; 25022; 25023; 25030; 25031; 25032; 25033; 25040; 25041; 25042; 25043; 25050; 25051; 25052; 25053; 25060; 25061; 25062; 25063; 25070; 25071; 25072; 25073; 25080; 25081; 25082; 25083; 25090; 25091; 25092; 25093; 3572; 36201; 36202; 36203; 36204; 36205; 36206; 36207; 36641; E0821; E0822; E0829; E08311; E08319; E083211; E083212; E083213; E083219; E08329; E083291; E083292; E083293; E083299; E083311; E083312; E083313; E083319; E083391; E083392; E083393; E083399; E083411; E083412; E083413; E083419; E083491; E083492; E083493; E083499; E083511; E083512; E083513; E083519; E083521; E083522; E083523; E083529; E083531; E083532; E083533; E083539; E083541; E083542; E083543; E083549; E083551; E083552; E083553; E083559; E083591; E083592; E083593; E083599; E0836; E0837X1; E0837X2; E0837X3; E0837X9; E0839; E0840; E0841; E0842; E0843; E0844; E0849; E0851; E0852; E0859; E08610; E08618; E08620; E08621; E08622; E08628; E08630; E08638; E08641; E08649; E0865; E0869; E088; E089; E0900; E0901; E0910; E0911; E0921; E0922; E0929; E09311; E09319; E093211; E093212; E093213; E093219; E093291; E093292; E093293; E093299; E09331; E093311; E093312; E093313; E093319; E093391; E093392; E093393; E093399; E093411; E093412; E093413; E093419; E093491; E093492; E093493; E093499; E093511; E093512; E093513; E093519; E093521; E093522; E093523; E093529; E093531; E093532; E093533; E093539; E093541; E093542; E093543; E093549; E093551; E093552; E093553; E093559; E09359; E093591; E093592; E093593; E093599; E0936; E0937X1; E0937X2; E0937X3; E0937X9; E0939; E0940; E0941; E0942; E0943; E0944; E0949; E0951; E0952; E0959; E09610; E09618; E09620; E09621; E09622; E09628; E09630; E09638; E09641; E09649; E0965; E0969; E098; E1021; E1022; E1029; E10311; E10319; E10321; E103211; E103212; E103213; E103219; E10329; E103291; E103292; E103293; E103299; E10331; E103311; E103312; E103313; E103319; E10339; E103391; E103392; E103393; E103399; E10341; E103411; E103412; E103413; E103419; E10349; E103491; E103492; E103493; E103499; E10351; E103511; E103512; E103513; E103519; E103521; E103522; E103523; E103529; E103531; E103532; E103533; E103539; E103541; E103542; E103543; E103549; E103551; E103552; E103553; E103559; E10359; E103591; E103592; E103593; E103599; E1036; E1037X1; E1037X2; E1037X3; E1037X9; E1039; E1040; E1041; E1042; E1043; E1044; E1049; E1051; E1052; E1059; E10610; E10618; E10620; E10621; E10622; E10628; E10630; E10638; E10641; E10649; E1065; E1069; E108; E1121; E1122; E1129; E11311; E11319; E11321; E113211; E113212; E113213; E113219; E11329; E113291; E113292; E113293; E113299; E11331; E113311; E113312; E113313; E113319; E11339; E113391; E113392; E113393; E113399; E11341; E113411; E113412; E113413; E113419; E11349; E113491; E113492; E113493; E113499; E11351; E113511; E113512; E113513; E113519; E113521; E113522; E113523; E113529; E113531; E113532; E113533; E113539; E113541; E113542; E113543; E113549; E113551; E113552; E113553; E113559; E11359; E113591; E113592; E113593; E113599; E1136; E1137X1; E1137X2; E1137X3; E1137X9; E1139; E1140; E1141; E1142; E1143; E1144; E1149; E1151; E1152; E1159; E11610; E11618; E11620; E11621; E11622; E11628; E11630; E11638; E11641; E11649; E1165; E1169; E118; E1321; E1322; E1329; E13311; E13319; E13321; E133211; E133212; E133213; E133219; E13329; E133291; E133292; E133293; E133299; E13331; E133311; E133312; E133313; E133319; E13339; E133391; E133392; E133393; E133399; E13341; E133411; E133412; E133413; E133419; E13349; E133491; E133492; E133493; E133499; E13351; E133511; E133512; E133513; E133519; E133521; E133522; E133523; E133529; E133531; E133532; E133533; E133539; E133541; E133542; E133543; E133549; E133551; E133552; E133553; E133559; E133591; E133592; E133593; E133599; E1336; E1337X1; E1337X2; E1337X3; E1337X9; E1339; E1340; E1341; E1342; E1343; E1344; E1349; E1351; E1352; E1359; E13610; E13618; E13620; E13621; E13622; E13628; E13630; E13638; E13641; E13649; E1365; E1369; E138 |
| Myocardial Infarction | I2101; I2102; I2109; I2111; I2119; I2121; I2129; I213; I214; I219 |
| Coronary Artery Disease | 41000; 41001; 41002; 41010; 41011; 41012; 41020; 41021; 41022; 41030; 41031; 41032; 41040; 41041; 41042; 41050; 41051; 41052; 41060; 41061; 41062; 41070; 41071; 41072; 41080; 41081; 41082; 41090; 41091; 41092; 4110; 4111; 41181; 41189; 412; 41400; 41401; 41402; 41403; 41404; 41405; 41406; 41407; 41410; 41411; 41412; 41419; 4142; 4143; 4144; 4148; 4149; 4292; 42979; I200; I2101; I2102; I2109; I2111; I2119; I2121; I2129; I213; I214; I219; I21A1; I21A9; I220; I221; I222; I228; I229; I230; I231; I232; I233; I234; I235; I236; I237; I238; I240; I241; I248; I249; I2510; I25111; I25118; I25119; I252; I253; I2541; I2542; I255; I256; I25700; I25701; I25708; I25709; I25710; I25711; I25718; I25719; I25720; I25721; I25728; I25729; I25730; I25731; I25738; I25739; I25750; I25751; I25758; I25759; I25760; I25761; I25768; I25769; I25790; I25791; I25798; I25799; I25810; I25811; I25812; I2582; I2583; I2584; I2589; I259 |
| Cerebrovascular Disease or  Ischemic Stroke | 1671; 43301; 43311; 43321; 43331; 43381; 43391; 43401; 43411; 43491; I6300; I63011; I63012; I63013; I63019; I6302; I63031; I63032; I63033; I63039; I6309; I6310; I63111; I63112; I63113; I63119; I6312; I63131; I63132; I63133; I63139; I6319; I6320; I63211; I63212; I63213; I63219; I6322; I63231; I63232; I63233; I63239; I6329; I6330; I63311; I63312; I63313; I63319; I63321; I63322; I63323; I63329; I63331; I63332; I63333; I63339; I63341; I63342; I63343; I63349; I6339; I6340; I63411; I63412; I63413; I63419; I63421; I63422; I63423; I63429; I63431; I63432; I63433; I63439; I63441; I63442; I63443; I63449; I6349; I6350; I63511; I63512; I63513; I63519; I63521; I63522; I63523; I63529; I63531; I63532; I63533; I63539; I63541; I63542; I63543; I63549; I6359; I636; I638; I6381; I6389; I639 |
| Transient Ischemic Attack | 4350; 4351;4352;4353;4358;4359;4377; G450; G451; G452; G453; G454; G458; G459 |
| Peripheral Arterial Disease | 34839; 43310; 4400; 4401; 44020; 44021; 44022; 44023; 44024; 44029; 44030; 44031; 44032; 4404; 4408; 4409; 44100; 44101; 44102; 44103; 4411; 4412; 4413; 4414; 4415; 4416; 4417; 4419; 4420; 4421; 4422; 4423; 44281; 44282; 44283; 44284; 44289; 4429; 4431; 44321; 44322; 44323; 44324; 44329; 44382; 44389; 4439; 44401; 44409; 4441; 44421; 44422; 44481; 44489; 4449; 4470; 4471; 4472; 4473; 4474; 4475; 4476; 44770; 44771; 44772; 44773; 4478; 4479; 5571; 5579; I700; I701; I70201; I70202; I70203; I70208; I70209; I70211; I70212; I70213; I70218; I70219; I70221; I70222; I70223; I70228; I70229; I70231; I70232; I70233; I70234; I70235; I70238; I70239; I70241; I70242; I70243; I70244; I70245; I70248; I70249; I7025; I70261; I70262; I70263; I70268; I70269; I70291; I70292; I70293; I70298; I70299; I70301; I70302; I70303; I70308; I70309; I70311; I70312; I70313; I70318; I70319; I70321; I70322; I70323; I70328; I70329; I70331; I70332; I70333; I70334; I70335; I70338; I70339; I70341; I70342; I70343; I70344; I70345; I70348; I70349; I7035; I70361; I70362; I70363; I70368; I70369; I70391; I70392; I70393; I70398; I70399; I70401; I70402; I70403; I70408; I70409; I70411; I70412; I70413; I70418; I70419; I70421; I70422; I70423; I70428; I70429; I70431; I70432; I70433; I70434; I70435; I70438; I70439; I70441; I70442; I70443; I70444; I70445; I70448; I70449; I7045; I70461; I70462; I70463; I70468; I70469; I70491; I70492; I70493; I70498; I70499; I70501; I70502; I70503; I70508; I70509; I70511; I70512; I70513; I70518; I70519; I70521; I70522; I70523; I70528; I70529; I70531; I70532; I70533; I70534; I70535; I70538; I70539; I70541; I70542; I70543; I70544; I70545; I70548; I70549; I7055; I70561; I70562; I70563; I70568; I70569; I70591; I70592; I70593; I70598; I70599; I70601; I70602; I70603; I70608; I70609; I70611; I70612; I70613; I70618; I70619; I70621; I70622; I70623; I70628; I70629; I70631; I70632; I70633; I70634; I70635; I70638; I70639; I70641; I70642; I70643; I70644; I70645; I70648; I70649; I7065; I70661; I70662; I70663; I70668; I70669; I70691; I70692; I70693; I70698; I70699; I70701; I70702; I70703; I70708; I70709; I70711; I70712; I70713; I70718; I70719; I70721; I70722; I70723; I70728; I70729; I70731; I70732; I70733; I70734; I70735; I70738; I70739; I70741; I70742; I70743; I70744; I70745; I70748; I70749; I7075; I70761; I70762; I70763; I70768; I70769; I70791; I70792; I70793; I70798; I70799; I708; I7090; I7091; I7092; I7100; I7101; I71010; I71011; I71012; I71019; I7102; I7103; I711; I7110; I7111; I7112; I7113; I712; I7120; I7121; I7122; I7123; I713; I7130; I7131; I7132; I7133; I714; I7140; I7141; I7142; I7143; I715; I7150; I7151; I7152; I716; I7160; I7161; I7162; I718; I719; I720; I721; I722; I723; I724; I725; I726; I728; I729; I7301; I731; I7381; I7389; I739; I7401; I7409; I7410; I7411; I7419; I742; I743; I744; I745; I748; I749; I770; I771; I772; I773; I774; I775; I776; I7770; I7771; I7772; I7773; I7774; I7775; I7776; I7777; I7779; I77810; I77811; I77812; I77819; I7782; I7789; I779; K551; K558; K559; M318; M319 |
| Heart Failure | I0981; I110; I130; I132; I501; I5020; I5021; I5022; I5023; I5030; I5031; I5032; I5033; I5040; I5041; I5042; I5043; I50810; I50811; I50812; I50813; I50814; I5082; I5083; I5084; I5089; I509; I5181; I97130; I97131; O29121; O29122; O29123; O29129; R570; Z95811; Z95812 |
| Moderate to Severe Chronic Kidney Disease | N183; N1830; N1831; N1832; N189; N184; N185; N186; N189; Z4901; Z4902; Z4931; Z4932; Z9115; Z940; Z992; 5853; 5854; 5855 |
| Atrial Fibrillation | 42731; 42732; I480; I481; I4811; I4819; I482; I4820; I4821; I483; I484; I4891; I4892 |
| Mild Liver disease | 701;7030;7031;7032;7033;7051;7052;7053;7054;7059;7070;709;9162;952;953;5710;5711;5712;5713;57140;57141;57142;57149;5715;5716;5718;5719;5720;5728;5730;5731;5732;5733;5734;5735;5738;5739;A5145;A5274;B180;B181;B182;B188;B189;B1910;B1920;B199;B251;B581;K700;K7010;K7011;K702;K7030;K7031;K709;K713;K714;K7150;K7151;K716;K717;K718;K730;K731;K732;K738;K739;K740;K7400;K7401;K7402;K741;K742;K743;K744;K745;K7460;K7469;K751;K752;K753;K754;K7581;K7589;K759;K760;K761;K762;K763;K764;K7681;K7682;K7689;K769;K77 |
| Severe Liver disease | 700; 7020; 7021; 7022; 7023; 7041; 7043; 7044; 7049; 706; 7071; 4560; 4561; 45620; 45621; 4568; 570; 5721; 5722; 5723; 5724; 99749; B190; B1911; B1921; I8500; I8501; I8510; I8511; I864; K7040; K7041; K7210; K7211; K7290; K7291; K765; K766; K767; K9182; V427; Z944 |
| Valvular heart disease | 3940; 3941; 3942; 3949; 3950; 3951; 3952; 3959; 3960; 3961; 3962; 3963; 3968; 3969; 3970; 3971; 3979; 39890; 39891; 39899; 4240; 4241; 4242; 4243; 74600; 74601; 74602; 74609; 7461; 7462; 7463; 7464; 7465; 7466; 7467; 99602; 99671; I050; I051; I052; I058; I059; I060; I061; I062; I068; I069; I070; I071; I072; I078; I079; I080; I081; I082; I083; I088; I089; I091; I0981; I0989; I099; I340; I341; I342; I348; I349; I350; I351; I352; I358; I359; I360; I361; I362; I368; I369; I370; I371; I372; I378; I379; Q220; Q221; Q222; Q223; Q224; Q225; Q226; Q228; Q229; Q230; Q231; Q232; Q233; Q234; Q238; Q239; T8201XA; T8201XD; T8201XS; T8202XA; T8202XD; T8202XS; T8203XA; T8203XD; T8203XS; T8209XA; T8209XD; T8209XS; T82221A; T82221D; T82221S; T82222A; T82222D; T82222S; T82223A; T82223D; T82223S; T82228A; T82228D; T82228S; T826XXA; T826XXD; T826XXS; V422; V433; Z952; Z953; Z954 |
| Asthma | 49300; 49301; 49302; 49310; 49311; 49312; 49320; 49321; 49322; 49381; 49382; 49390; 49391; 49392; J4520; J4521; J4522; J4530; J4531; J4532; J4540; J4541; J4542; J4550; J4551; J4552; J45901; J45902; J45909; J45990; J45991; J45998; |
| COPD | 490; 4910; 4911; 4912; 49120; 49121; 49122; 4918; 4919; 4920; 4928; 494; 4940; 4941; 496; J40; J410; J411; J418; J42; J430; J431; J432; J438; J439; J440; J441; J449; J470; J471; J479 |
| Hyperlipidemia | 2720;2721;2722;2723;2724;2725;2726;2727;2728;2729; E780; E7800; E7801; E781; E782; E783; E784; E7841; E7849; E785; E786; E7870; E7871; E7872; E7879; E7881; E7889; E789 |
| Hypertension | 4010; 4011; 4019; 40200; 40201; 40210; 40211; 40290; 40291; 40300; 40301; 40310; 40311; 40390; 40391; 40400; 40401; 40402; 40403; 40410; 40411; 40412; 40413; 40490; 40491; 40492; 40493; 40501; 40509; 40511; 40519; 40591; 40599; I10; I110; I119; I120; I129; I130; I1310; I1311; I132; I150; I151; I152; I158; I159; I160; I161; I169 |
|  | **LOINC codes** |
| LDL | 13457-7;18261-8;18262-6;2089-1;2090-9;35198-1;49132-4;55440-2;96259-7;96597-0 |
| Blood Urea Nitrogen | 3094-0;11064-3;11065-0;12961-9;12962-7;12964-3;12965-0;12966-8;20977-5;6299-2;3091-6;35234-4;42571-0;72270-2;93975-1;94003-1;94012-2 |
| Hemoglobin | 17855-8;17856-6;43150-2;4548-4;4549-2;54039-3;55454-3;59261-8;62388-4;67761-7;71875-9;74246-0;75862-3;86910-7;96070-8;96595-4;62853-7;62854-5 |
| Creatinine | 11041-1; 11042-9; 16188-5; 16189-3; 21232-4; 2160-0; 35203-9; 38483-4; 39955-0; 39956-8; 39957-6; 39958-4; 39959-2; 39960-0; 39961-8; 39962-6; 39963-4; 39964-2; 39965-9; 39966-7; 39967-5; 39968-3; 39969-1; 39970-9; 39971-7; 39972-5; 39973-3; 39974-1; 39975-8; 39976-6; 40248-7; 40249-5; 40250-3; 40251-1; 40252-9; 40253-7; 40254-5; 40255-2; 40256-0; 40257-8; 40258-6; 44784-7; 72271-0; 93999-1; 94008-0; 96590-5 |
| hsCRP | 30522-7; 71426-1; 76486-0 |
| Brain Natriuretic peptide | 30934-4; 35257-5; 42637-9; 47092-2; 3094-0; 11064-3; 11065-0; 12961-9; 12962-7; 12964-3; 12965-0; 12966-8; 20977-5; 6299-2; 3091-6; 35234-4; 42571-0; 72270-2; 93975-1; 94003-1; 94012-2; |
|  | **Procedure codes** |
| Peripheral Arterial Revascularization | 0238T; 35381; 35450; 35452; 35454; 35456; 35459; 35470; 35472; 35473; 35474; 35481; 35482; 35483; 35485; 35491; 35492; 35493; 35495; 35521; 35533; 35537; 35538; 35539; 35540; 35541; 35546; 35548; 35549; 35551; 35556; 35558; 35563; 35565; 35566; 35571; 35582; 35583; 35585; 35587; 35621; 35623; 35637; 35638; 35641; 35646; 35647; 35651; 35654; 35656; 35661; 35663; 35665; 35666; 35671; 35879; 35881; 35883; 35884; 36140; 36200; 36245; 36246; 36247; 36248; 37184; 37185; 37186; 37205; 37206; 37207; 37208; 37220; 37221; 37222; 37223; 37224; 37225; 37226; 37227; 37228; 37229; 37230; 37231; 37232; 37233; 37234; 37235; 37236; 37237; 37238; 37239; 0045; 0046; 0047; 0048; 0055; 0060; 1756; 3808; 3818; 3838; 3848; 3925; 3929; 3949; 3950; 3956; 3957; 3958; 3990; 047C04Z; 047C0DZ; 047C0ZZ; 047C34Z; 047C3DZ; 047C3ZZ; 047C44Z; 047C4DZ; 047C4ZZ; 047D04Z; 047D0DZ; 047D0ZZ; 047D34Z; 047D3DZ; 047D3ZZ; 047D44Z; 047D4DZ; 047D4ZZ; 047E04Z; 047E0DZ; 047E0ZZ; 047E34Z; 047E3DZ; 047E3ZZ; 047E44Z; 047E4DZ; 047E4ZZ; 047F04Z; 047F0DZ; 047F0ZZ; 047F34Z; 047F3DZ; 047F3ZZ; 047F44Z; 047F4DZ; 047F4ZZ; 047H04Z; 047H0DZ; 047H0ZZ; 047H34Z; 047H3DZ; 047H3ZZ; 047H44Z; 047H4DZ; 047H4ZZ; 047J04Z; 047J0DZ; 047J0ZZ; 047J34Z; 047J3DZ; 047J3ZZ; 047J44Z; 047J4DZ; 047J4ZZ; 047K041; 047K04Z; 047K0D1; 047K0DZ; 047K0Z1; 047K0ZZ; 047K341; 047K34Z; 047K3D1; 047K3DZ; 047K3Z1; 047K3ZZ; 047K441; 047K44Z; 047K4D1; 047K4DZ; 047K4Z1; 047K4ZZ; 047L041; 047L04Z; 047L0D1; 047L0DZ; 047L0Z1; 047L0ZZ; 047L341; 047L34Z; 047L3D1; 047L3DZ; 047L3Z1; 047L3ZZ; 047L441; 047L44Z; 047L4D1; 047L4DZ; 047L4Z1; 047L4ZZ; 047M041; 047M04Z; 047M0D1; 047M0DZ; 047M0Z1; 047M0ZZ; 047M341; 047M34Z; 047M3D1; 047M3DZ; 047M3Z1; 047M3ZZ; 047M441; 047M44Z; 047M4D1; 047M4DZ; 047M4Z1; 047M4ZZ; 047N041; 047N04Z; 047N0D1; 047N0DZ; 047N0Z1; 047N0ZZ; 047N341; 047N34Z; 047N3D1; 047N3DZ; 047N3Z1; 047N3ZZ; 047N441; 047N44Z; 047N4D1; 047N4DZ; 047N4Z1; 047N4ZZ; 047P04Z; 047P0DZ; 047P0ZZ; 047P34Z; 047P3DZ; 047P3ZZ; 047P44Z; 047P4DZ; 047P4ZZ; 047Q04Z; 047Q0DZ; 047Q0ZZ; 047Q34Z; 047Q3DZ; 047Q3ZZ; 047Q44Z; 047Q4DZ; 047Q4ZZ; 047R04Z; 047R0DZ; 047R0ZZ; 047R34Z; 047R3DZ; 047R3ZZ; 047R44Z; 047R4DZ; 047R4ZZ; 047S04Z; 047S0DZ; 047S0ZZ; 047S34Z; 047S3DZ; 047S3ZZ; 047S44Z; 047S4DZ; 047S4ZZ; 047T04Z; 047T0DZ; 047T0ZZ; 047T34Z; 047T3DZ; 047T3ZZ; 047T44Z; 047T4DZ; 047T4ZZ; 047U04Z; 047U0DZ; 047U0ZZ; 047U34Z; 047U3DZ; 047U3ZZ; 047U44Z; 047U4DZ; 047U4ZZ; 047V04Z; 047V0DZ; 047V0ZZ; 047V34Z; 047V3DZ; 047V3ZZ; 047V44Z; 047V4DZ; 047V4ZZ; 047W04Z; 047W0DZ; 047W0ZZ; 047W34Z; 047W3DZ; 047W3ZZ; 047W44Z; 047W4DZ; 047W4ZZ; 031209B; 031209C; 03120A6; 03120A7; 03120A8; 03120A9; 03120AB; 03120AC; 03120J6; 03120J7; 03120J8; 03120J9; 03120JB; 03120JC; 03120K6; 03120K7; 03120K8; 03120K9; 03120KB; 03120KC; 03120Z6; 03120Z7; 03120Z8; 03120Z9; 03120ZB; 03120ZC; 031309B; 031309C; 03130A6; 03130A7; 03130A8; 03130A9; 03130AB; 03130AC; 03130J6; 03130J7; 03130J8; 03130J9; 03130JB; 03130JC; 03130K6; 03130K7; 03130K8; 03130K9; 03130KB; 03130KC; 041009B; 041009C; 041009D; 041009F; 041009G; 041009H; 041009J; 041009K; 041009Q; 041009R; 04100A6; 04100A7; 04100A8; 04100A9; 04100AB; 04100AC; 04100AD; 04100AF; 04100AG; 04100AH; 04100AJ; 04100AK; 04100AQ; 04100AR; 04100J6; 04100J7; 04100J8; 04100J9; 04100JB; 04100JC; 04100JD; 04100JF; 04100JG; 04100JH; 04100JJ; 04100JK; 04100JQ; 04100JR; 04100K6; 04100K7; 04100K8; 04100K9; 04100KB; 04100KC; 04100KD; 04100KF; 04100KG; 04100KH; 04100KJ; 04100KK; 04100KQ; 04100KR; 04100Z6; 04100Z7; 04100Z8; 04100Z9; 04100ZB; 04100ZC; 04100ZD; 04100ZF; 04100ZG; 04100ZH; 04100ZJ; 04100ZK; 04100ZQ; 04100ZR; 041049B; 041049C; 041049D; 041049F; 041049G; 041049H; 041049J; 041049K; 041049Q; 041049R; 04104A6; 04104A7; 04104A8; 04104A9; 04104AB; 04104AC; 04104AD; 04104AF; 04104AG; 04104AH; 04104AJ; 04104AK; 04104AQ; 04104AR; 04104J6; 04104J7; 04104J8; 04104J9; 04104JB; 04104JC; 04104JD; 04104JF; 04104JG; 04104JH; 04104JJ; 04104JK; 04104JQ; 04104JR; 04104K6; 04104K7; 04104K8; 04104K9; 04104KB; 04104KC; 04104KD; 04104KF; 04104KG; 04104KH; 04104KJ; 04104KK; 04104KQ; 04104KR; 04104Z6; 04104Z7; 04104Z8; 04104Z9; 04104ZB; 04104ZC; 04104ZD; 04104ZF; 04104ZG; 04104ZH; 04104ZJ; 04104ZK; 04104ZQ; 04104ZR; 041C09H; 041C09J; 041C09K; 041C0AH; 041C0AJ; 041C0AK; 041C0JH; 041C0JJ; 041C0JK; 041C0KH; 041C0KJ; 041C0KK; 041C0ZH; 041C0ZJ; 041C0ZK; 041C49H; 041C49J; 041C49K; 041C4AH; 041C4AJ; 041C4AK; 041C4JH; 041C4JJ; 041C4JK; 041C4KH; 041C4KJ; 041C4KK; 041C4ZH; 041C4ZJ; 041C4ZK; 041D09H; 041D09J; 041D09K; 041D0AH; 041D0AJ; 041D0AK; 041D0JH; 041D0JJ; 041D0JK; 041D0KH; 041D0KJ; 041D0KK; 041D0ZH; 041D0ZJ; 041D0ZK; 041D49H; 041D49J; 041D49K; 041D4AH; 041D4AJ; 041D4AK; 041D4JH; 041D4JJ; 041D4JK; 041D4KH; 041D4KJ; 041D4KK; 041D4ZH; 041D4ZJ; 041D4ZK; 041E09H; 041E09J; 041E09K; 041E0AH; 041E0AJ; 041E0AK; 041E0JH; 041E0JJ; 041E0JK; 041E0KH; 041E0KJ; 041E0KK; 041E0ZH; 041E0ZJ; 041E0ZK; 041E49H; 041E49J; 041E49K; 041E4AH; 041E4AJ; 041E4AK; 041E4JH; 041E4JJ; 041E4JK; 041E4KH; 041E4KJ; 041E4KK; 041E4ZH; 041E4ZJ; 041E4ZK; 041F09H; 041F09J; 041F09K; 041F0AH; 041F0AJ; 041F0AK; 041F0JH; 041F0JJ; 041F0JK; 041F0KH; 041F0KJ; 041F0KK; 041F0ZH; 041F0ZJ; 041F0ZK; 041F49H; 041F49J; 041F49K; 041F4AH; 041F4AJ; 041F4AK; 041F4JH; 041F4JJ; 041F4JK; 041F4KH; 041F4KJ; 041F4KK; 041F4ZH; 041F4ZJ; 041F4ZK; 041H09H; 041H09J; 041H09K; 041H0AH; 041H0AJ; 041H0AK; 041H0JH; 041H0JJ; 041H0JK; 041H0KH; 041H0KJ; 041H0KK; 041H0ZH; 041H0ZJ; 041H0ZK; 041H49H; 041H49J; 041H49K; 041H4AH; 041H4AJ; 041H4AK; 041H4JH; 041H4JJ; 041H4JK; 041H4KH; 041H4KJ; 041H4KK; 041H4ZH; 041H4ZJ; 041H4ZK; 041J09H; 041J09J; 041J09K; 041J0AH; 041J0AJ; 041J0AK; 041J0JH; 041J0JJ; 041J0JK; 041J0KH; 041J0KJ; 041J0KK; 041J0ZH; 041J0ZJ; 041J0ZK; 041J49H; 041J49J; 041J49K; 041J4AH; 041J4AJ; 041J4AK; 041J4JH; 041J4JJ; 041J4JK; 041J4KH; 041J4KJ; 041J4KK; 041J4ZH; 041J4ZJ; 041J4ZK; 041K09H; 041K09J; 041K09K; 041K09L; 041K09M; 041K09N; 041K09P; 041K09Q; 041K09S; 041K0AH; 041K0AJ; 041K0AK; 041K0AL; 041K0AM; 041K0AN; 041K0AP; 041K0AQ; 041K0AS; 041K0JH; 041K0JJ; 041K0JK; 041K0JL; 041K0JM; 041K0JN; 041K0JP; 041K0JQ; 041K0JS; 041K0KH; 041K0KJ; 041K0KK; 041K0KL; 041K0KM; 041K0KN; 041K0KP; 041K0KQ; 041K0KS; 041K0ZH; 041K0ZJ; 041K0ZK; 041K0ZL; 041K0ZM; 041K0ZN; 041K0ZP; 041K0ZQ; 041K0ZS; 041K49H; 041K49J; 041K49K; 041K49L; 041K49M; 041K49N; 041K49P; 041K49Q; 041K49S; 041K4AH; 041K4AJ; 041K4AK; 041K4AL; 041K4AM; 041K4AN; 041K4AP; 041K4AQ; 041K4AS; 041K4JH; 041K4JJ; 041K4JK; 041K4JL; 041K4JM; 041K4JN; 041K4JP; 041K4JQ; 041K4JS; 041K4KH; 041K4KJ; 041K4KK; 041K4KL; 041K4KM; 041K4KN; 041K4KP; 041K4KQ; 041K4KS; 041K4ZH; 041K4ZJ; 041K4ZK; 041K4ZL; 041K4ZM; 041K4ZN; 041K4ZP; 041K4ZQ; 041K4ZS; 041L09H; 041L09J; 041L09K; 041L09L; 041L09M; 041L09N; 041L09P; 041L09Q; 041L09S; 041L0AH; 041L0AJ; 041L0AK; 041L0AL; 041L0AM; 041L0AN; 041L0AP; 041L0AQ; 041L0AS; 041L0JH; 041L0JJ; 041L0JK; 041L0JL; 041L0JM; 041L0JN; 041L0JP; 041L0JQ; 041L0JS; 041L0KH; 041L0KJ; 041L0KK; 041L0KL; 041L0KM; 041L0KN; 041L0KP; 041L0KQ; 041L0KS; 041L0ZH; 041L0ZJ; 041L0ZK; 041L0ZL; 041L0ZM; 041L0ZN; 041L0ZP; 041L0ZQ; 041L0ZS; 041L49H; 041L49J; 041L49K; 041L49L; 041L49M; 041L49N; 041L49P; 041L49Q; 041L49S; 041L4AH; 041L4AJ; 041L4AK; 041L4AL; 041L4AM; 041L4AN; 041L4AP; 041L4AQ; 041L4AS; 041L4JH; 041L4JJ; 041L4JK; 041L4JL; 041L4JM; 041L4JN; 041L4JP; 041L4JQ; 041L4JS; 041L4KH; 041L4KJ; 041L4KK; 041L4KL; 041L4KM; 041L4KN; 041L4KP; 041L4KQ; 041L4KS; 041L4ZH; 041L4ZJ; 041L4ZK; 041L4ZL; 041L4ZM; 041L4ZN; 041L4ZP; 041L4ZQ; 041L4ZS; 041M09L; 041M09M; 041M09P; 041M09Q; 041M09S; 041M0AL; 041M0AM; 041M0AP; 041M0AQ; 041M0AS; 041M0JL; 041M0JM; 041M0JP; 041M0JQ; 041M0JS; 041M0KL; 041M0KM; 041M0KP; 041M0KQ; 041M0KS; 041M0ZL; 041M0ZM; 041M0ZP; 041M0ZQ; 041M0ZS; 041M49L; 041M49M; 041M49P; 041M49Q; 041M49S; 041M4AL; 041M4AM; 041M4AP; 041M4AQ; 041M4AS; 041M4JL; 041M4JM; 041M4JP; 041M4JQ; 041M4JS; 041M4KL; 041M4KM; 041M4KP; 041M4KQ; 041M4KS; 041M4ZL; 041M4ZM; 041M4ZP; 041M4ZQ; 041M4ZS; 041N09L; 041N09M; 041N09P; 041N09Q; 041N09S; 041N0AL; 041N0AM; 041N0AP; 041N0AQ; 041N0AS; 041N0JL; 041N0JM; 041N0JP; 041N0JQ; 041N0JS; 041N0KL; 041N0KM; 041N0KP; 041N0KQ; 041N0KS; 041N0ZL; 041N0ZM; 041N0ZP; 041N0ZQ; 041N0ZS; 041N49L; 041N49M; 041N49P; 041N49Q; 041N49S; 041N4AL; 041N4AM; 041N4AP; 041N4AQ; 041N4AS; 041N4JL; 041N4JM; 041N4JP; 041N4JQ; 041N4JS; 041N4KL; 041N4KM; 041N4KP; 041N4KQ; 041N4KS; 041N4ZL; 041N4ZM; 041N4ZP; 041N4ZQ; 041N4ZS; 04RK07Z; 04RK0JZ; 04RK0KZ; 04RK47Z; 04RK4JZ; 04RK4KZ; 04RL07Z; 04RL0JZ; 04RL0KZ; 04RL47Z; 04RL4JZ; 04RL4KZ; 04RM07Z; 04RM0JZ; 04RM0KZ; 04RM47Z; 04RM4JZ; 04RM4KZ; 04RN07Z; 04RN0JZ; 04RN0KZ; 04RN47Z; 04RN4JZ; 04RN4KZ; 04RP07Z; 04RP0JZ; 04RP0KZ; 04RP47Z; 04RP4JZ; 04RP4KZ; 04RQ07Z; 04RQ0JZ; 04RQ0KZ; 04RQ47Z; 04RQ4JZ; 04RQ4KZ; 04RR07Z; 04RR0JZ; 04RR0KZ; 04RR47Z; 04RR4JZ; 04RR4KZ; 04RS07Z; 04RS0JZ; 04RS0KZ; 04RS47Z; 04RS4JZ; 04RS4KZ; 04RT07Z; 04RT0JZ; 04RT0KZ; 04RT47Z; 04RT4JZ; 04RT4KZ; 04RU07Z; 04RU0JZ; 04RU0KZ; 04RU47Z; 04RU4JZ; 04RU4KZ; 04RV07Z; 04RV0JZ; 04RV0KZ; 04RV47Z; 04RV4JZ; 04RV4KZ; 04RW07Z; 04RW0JZ; 04RW0KZ; 04RW47Z; 04RW4JZ; 04RW4KZ; 04RY07Z; 04RY0JZ; 04RY0KZ; 04RY47Z; 04RY4JZ; 04RY4KZ; 04UC07Z; 04UC0JZ; 04UC0KZ; 04UC37Z; 04UC3JZ; 04UC3KZ; 04UC47Z; 04UC4JZ; 04UC4KZ; 04UD07Z; 04UD0JZ; 04UD0KZ; 04UD37Z; 04UD3JZ; 04UD3KZ; 04UD47Z; 04UD4JZ; 04UD4KZ; 04UE07Z; 04UE0JZ; 04UE0KZ; 04UE37Z; 04UE3JZ; 04UE3KZ; 04UE47Z; 04UE4JZ; 04UE4KZ; 04UF07Z; 04UF0JZ; 04UF0KZ; 04UF37Z; 04UF3JZ; 04UF3KZ; 04UF47Z; 04UF4JZ; 04UF4KZ; 04UH07Z; 04UH0JZ; 04UH0KZ; 04UH37Z; 04UH3JZ; 04UH3KZ; 04UH47Z; 04UH4JZ; 04UH4KZ; 04UJ07Z; 04UJ0JZ; 04UJ0KZ; 04UJ37Z; 04UJ3JZ; 04UJ3KZ; 04UJ47Z; 04UJ4JZ; 04UJ4KZ; 04UK07Z; 04UK0JZ; 04UK0KZ; 04UK37Z; 04UK3JZ; 04UK3KZ; 04UK47Z; 04UK4JZ; 04UK4KZ; 04UL07Z; 04UL0JZ; 04UL0KZ; 04UL37Z; 04UL3JZ; 04UL3KZ; 04UL47Z; 04UL4JZ; 04UL4KZ; 04UM07Z; 04UM0JZ; 04UM0KZ; 04UM37Z; 04UM3JZ; 04UM3KZ; 04UM47Z; 04UM4JZ; 04UM4KZ; 04UN07Z; 04UN0JZ; 04UN0KZ; 04UN37Z; 04UN3JZ; 04UN3KZ; 04UN47Z; 04UN4JZ; 04UN4KZ; 04UP07Z; 04UP0JZ; 04UP0KZ; 04UP37Z; 04UP3JZ; 04UP3KZ; 04UP47Z; 04UP4JZ; 04UP4KZ; 04UQ07Z; 04UQ0JZ; 04UQ0KZ; 04UQ37Z; 04UQ3JZ; 04UQ3KZ; 04UQ47Z; 04UQ4JZ; 04UQ4KZ; 04UR07Z; 04UR0JZ; 04UR0KZ; 04UR37Z; 04UR3JZ; 04UR3KZ; 04UR47Z; 04UR4JZ; 04UR4KZ; 04US07Z; 04US0JZ; 04US0KZ; 04US37Z; 04US3JZ; 04US3KZ; 04US47Z; 04US4JZ; 04US4KZ; 04UT07Z; 04UT0JZ; 04UT0KZ; 04UT37Z; 04UT3JZ; 04UT3KZ; 04UT47Z; 04UT4JZ; 04UT4KZ; 04UU07Z; 04UU0JZ; 04UU0KZ; 04UU37Z; 04UU3JZ; 04UU3KZ; 04UU47Z; 04UU4JZ; 04UU4KZ; 04UV07Z; 04UV0JZ; 04UV0KZ; 04UV37Z; 04UV3JZ; 04UV3KZ; 04UV47Z; 04UV4JZ; 04UV4KZ; 04UW07Z; 04UW0JZ; 04UW0KZ; 04UW37Z; 04UW3JZ; 04UW3KZ; 04UW47Z; 04UW4JZ; 04UW4KZ; 04UY07Z; 04UY0JZ; 04UY0KZ; 04UY37Z; 04UY3JZ; 04UY3KZ; 04UY47Z; 04UY4JZ; 04UY4KZ |

# **Supplement Table 2. Association Between hsCRP Quartiles and Adverse Cardiovascular Outcomes**

| **Outcomes** | **Model A**^†^ | | | **Model B**^‡^ | | |
| --- | --- | --- | --- | --- | --- | --- |
|  | **hsCRP Quantile 2 (hsCRP>1.905 and <=3.7)** | **hsCRP Quantile 3 (hsCRP>3.7 and <=6.2)** | **hsCRP Quantile 4 (Highest hsCRP) (hsCRP>6.2 and <=10)** | **hsCRP Quantile 2 (hsCRP>1.905 and <=3.7)** | **hsCRP Quantile 3 (hsCRP>3.7 and <=6.2)** | **hsCRP Quantile 4 (Highest hsCRP) (hsCRP>6.2 and <=10)** |
| 3-point MACE^§^ | 1.213* | 1.350* | 1.583* | 1.175* | 1.251* | 1.433* |
|  | (1.157 - 1.271) | (1.288 - 1.414) | (1.512 - 1.657) | (1.121 - 1.232) | (1.193 - 1.312) | (1.368 - 1.502) |
| All-cause death | 1.191* | 1.357* | 1.581* | 1.165* | 1.268* | 1.458* |
|  | (1.131 - 1.254) | (1.289 - 1.428) | (1.504 - 1.662) | (1.106 - 1.228) | (1.204 - 1.336) | (1.386 - 1.535) |
| Cardiovascular death | 1.161* | 1.293* | 1.510* | 1.135* | 1.208* | 1.387* |
|  | (1.071 - 1.258) | (1.194 - 1.401) | (1.396 - 1.633) | (1.047 - 1.231) | (1.114 - 1.310) | (1.281 - 1.503) |
| Heart failure hospitalization | 1.278* | 1.277* | 1.699* | 1.187* | 1.130* | 1.439* |
|  | (1.156 - 1.413) | (1.153 - 1.414) | (1.542 - 1.873) | (1.073 - 1.313) | (1.019 - 1.253) | (1.303 - 1.590) |
| ED visit for heart failure | 1.253* | 1.276* | 1.487* | 1.184* | 1.168* | 1.318* |
|  | (1.110 - 1.415) | (1.129 - 1.443) | (1.318 - 1.677) | (1.048 - 1.338) | (1.032 - 1.323) | (1.165 - 1.491) |
| Abbreviations: ACD: All-cause death; CVD: cardiovascular death; hsCRP: high-sensitivity C-reactive protein; MACE: Major adverse cardiovascular event * Denotes p<0.05 ^†^ Model A adjusted for age, sex, and year of hsCRP measurement. ^‡^ Model B adjusted for age, sex, year of hsCRP measurement, Charlson comorbidity index, prior MI, prior coronary revascularization, smoking status, body mass index, GFR, lipid-lowering therapies, antiplatelets, anticoagulants, angiotensin-converting enzyme inhibitors/angiotensin receptor blockers, SGLT-2i, and GLP-1 RA ^§^ 3-point MACE includes all-cause death, Heart failure hospitalization, ED visit for heart failure | | | | | | |

| **Supplement Table 3. Rates of Adverse Cardiovascular Outcomes among Veterans with and without SI Excluding Individuals with Systemic Autoimmune Conditions** | | | |  |
| --- | --- | --- | --- | --- |
| **Clinical Outcome** | **hsCRP≥2 and ≤ 10 (N=21,004)** | **hsCRP<2 (N=7,521)** | **p-value** |  |
| 3-point MACE* | 15.12 | 11.73 | <.0001 |  |
|  | (14.86 - 15.39) | (11.35 - 12.11) |  |  |
| All-cause death | 12.59 | 9.84 | <.0001 |  |
|  | (12.36 - 12.83) | (9.51 - 10.19) |  |  |
| Cardiovascular death | 5.06 | 4.11 | <.0001 |  |
|  | (4.9 - 5.22) | (3.89 - 4.35) |  |  |
| Heart failure hospitalization | 2.73 | 2.05 | <.0001 |  |
|  | (2.62 - 2.85) | (1.9 - 2.22) |  |  |
| ED visit for heart failure | 1.82 | 1.45 | <.0001 |  |
|  | (1.73 - 1.91) | (1.33 - 1.59) |  |  |
| Abbreviations: ACD: All-cause death; CVD: cardiovascular death; ED: Emergency department; hsCRP: high-sensitivity C-reactive protein; MACE: Major adverse cardiovascular events; SI: systemic inflammation * 3-point MACE includes all-cause death, Heart failure hospitalization, ED visit for heart failure | | | |  |
|  | | | |  |
|  |  |  |  |  |
|  |  |  |  |  |

# **Supplement Table 4. Rates of Adverse Cardiovascular Outcomes with and without SI Including hsCRP>10mg/L**

| **Outcomes** | **hsCRP≥2 (n=41,290)** | **hsCRP<2 (n=8,696)** | **p-value** |
| --- | --- | --- | --- |
| 3-point MACE* | 17.92 | 11.78 | <.0001 |
|  | (17.7 - 18.14) | (11.43 - 12.14) |  |
| All-cause death | 15.12 | 9.88 | <.0001 |
|  | (14.93 - 15.31) | (9.56 - 10.2) |  |
| Cardiovascular death | 5.94 | 4.11 | <.0001 |
|  | (5.81 - 6.07) | (3.9 - 4.34) |  |
| Heart failure hospitalization | 3.05 | 2.05 | <.0001 |
|  | (2.96 - 3.14) | (1.91 - 2.21) |  |
| ED visit for heart failure | 1.94 | 1.45 | <.0001 |
|  | (1.87 - 2.01) | (1.33 - 1.58) |  |

Abbreviations: ACD: All-cause death; CVD: cardiovascular death; ED: Emergency department.

hsCRP: high-sensitivity C-reactive protein; MACE: Major adverse cardiovascular events

* 3-point MACE includes all-cause death, Heart failure hospitalization, ED visit for heart failure

# **Supplement Table 5: Hazard Ratio of Systemic Inflammation (hsCRP≥2) In Primary Analysis and Complete Case Analysis of patients with vs. without Systemic Inflammation***

| **Outcomes** |  | **Hazard Ratio with SI** | | |
| --- | --- | --- | --- | --- |
|  | **Complete Case Without Missing BMI or eGFR (n=31,837)** | | **Complete Case Without Missing BMI, eGFR, or EF**  **(n=27,779)** | **Primary Analysis**  **(n=34,048)** |
| 3-point MACE^†^ | 1.28 | | 1.27 | 1.28 |
|  | (1.23 - 1.32) | | (1.22 - 1.32) | (1.24 - 1.34) |
| All-cause death | 1.29 | | 1.29 | 1.29 |
|  | (1.24 - 1.34) | | (1.24 - 1.34) | (1.25 - 1.34) |
| Cardiovascular death | 1.22 | | 1.22 | 1.23 |
|  | (1.14 - 1.30) | | (1.14 – 1.30) | (1.16 - 1.31) |
| Heart failure hospitalization | 1.25 | | 1.25 | 1.24 |
|  | (1.15 - 1.37) | | (1.15 – 1.37) | (1.14 - 1.35) |
| ED visit for heart failure | 1.22 | | 1.22 | 1.22 |
|  | (1.09 - 1.35) | | (1.09 – 1.35) | (1.10 - 1.35) |

Abbreviations: ED: Emergency Department; hsCRP: high-sensitivity C-reactive protein; MACE: major adverse cardiovascular events; SI: systemic inflammation.

^*^Adjusted for age, sex, year of hsCRP measurement, Charlson comorbidity index, prior MI, prior coronary revascularization, smoking status, body mass index, GFR, lipid-lowering therapies, antiplatelets, anticoagulants, angiotensin-converting enzyme inhibitors/angiotensin receptor blockers, SGLT-2i, and GLP-1 RA.

^†^3-point MACE includes all-cause death, heart failure hospitalization, and ED visit for heart failure

# **Supplement Table 6. Annual Rates of Acute Care Utilization among Veterans with and without SI***

| **Outcomes** | **Overall** | **hsCRP < 2mg/L** | **hsCRP ≥2 and ≤ 10mg/L** | **p-value** |
| --- | --- | --- | --- | --- |
| All cause hospitalization | 1.11 (1.10 - 1.11) | 0.93 (0.91 -0.93) | 1.17 (1.16 - 1.18) | <.0001 |
| Cardiovascular hospitalization | 0.30 (0.30-0.31) | 0.26 (0.25 - 0.26) | 0.32 (0.31 - 0.32) | <.0001 |
| Heart failure hospitalization | 0.05 (0.04 - 0.05) | 0.04 (0.03 - 0.04) | 0.05 (0.05 - 0.05) | <.0001 |
| Emergency department visits for heart failure | 0.025 (0.02 - 0.02) | 0.02 (0.01 - 0.02) | 0.03 (0.02 - 0.02) | <.0001 |
| Emergency department visits | 2.02 (2.01-2.02) | 1.80 (1.78 - 1.81) | 2.09 (2.08 - 2.1) | <.0001 |

Abbreviations: hsCRP: high-sensitivity C-reactive protein; SI: systemic inflammation

^*^Indicates p<0.05 for incidence rate ratio between SI (hsCRP≥2mg/L and <2mg/L) and the outcome.

# **Supplement Table 7. Marginal Cost of SI among Veterans with and without SI Using Tobit Model***

|  | **1-year Follow-up** | | |
| --- | --- | --- | --- |
| **Outcomes** |  | **Model B with Gamma Model and Two-Stage Model*** | **Model B with Tobit** |
| Total |  | 9,112 | 9,210 |
|  |  | (6,135 - 12,089) | (1,001 - 17,419) |
| Inpatient |  | 5,648 | 15,179 |
|  |  | (4,064 - 7,231) | (11,886 - 18,471) |
| Cardiovascular |  | 1,624 | 1,644 |
|  |  | (572 - 2,676) | (511 - 2,778) |
| Outpatient |  | 2,092 | 2,018 |
|  |  | (1,288 - 2,896) | (1,143 – 2,892) |
| Heart Failure |  | 1,208 | 3,317 |
|  |  | (842.9 - 1,574) | (2,472 – 4,164) |
| Pharmacy |  | 710 | 2,067 |
|  |  | (-279 - 1,700) | (-5,917 – 10,055) |

Abbreviations: hsCRP: high-sensitivity C-reactive protein; SI: systemic inflammation

^*^ Model B adjusted for age, sex, year of hsCRP measurement, Charlson comorbidity index, prior MI, prior coronary revascularization, smoking status, body mass index, GFR, lipid-lowering therapies, antiplatelets, anticoagulants, angiotensin-converting enzyme inhibitors/angiotensin receptor blockers, SGLT-2i, and GLP-1 RA. Two-stage model used for inpatient, cardiovascular, and heart failure costs.

# **Supplement Figure 1. Association Between hsCRP Modeled as a Spline and Composite MACE Outcome**

**REFERENCES**

1. Sandhu AT, Zheng J, Skye M, Heidenreich PA. Updating the Accuracy of Administrative Claims for Identifying Left Ventricular Ejection Fraction Among Patients With Heart Failure. Circ Cardiovasc Qual Outcomes. 2023 Apr;16(4):e008919.
